# Air–Sea Exchange and Atmospheric Deposition of Phthalate Esters in the South China Sea

**DOI:** 10.1021/acs.est.2c09426

**Published:** 2023-07-17

**Authors:** Lijie Mi, Zhiyong Xie, Weihai Xu, Joanna J. Waniek, Thomas Pohlmann, Wenying Mi

**Affiliations:** † Institute of Coastal Environmental Chemistry, Helmholtz-Zentrum Hereon, Geesthacht 21502, Germany; ‡ Institute of Oceanography, University of Hamburg, Hamburg 20146, Germany; § Key Laboratory of Ocean and Marginal Sea Geology, South China Sea Institute of Oceanology, Chinese Academy of Sciences, Guangzhou 510301, China; ∥ Department of Marine Chemistry, Leibniz Institute for Baltic Sea Research Warnemünde, Rostock 18119, Germany; ⊥ MINJIE Institute of Environmental Science and Health Research, Geesthacht 21502, Germany

**Keywords:** phthalate ester, emerging organic contaminants, air−sea exchange, atmospheric deposition, long-range transport, South China Sea

## Abstract

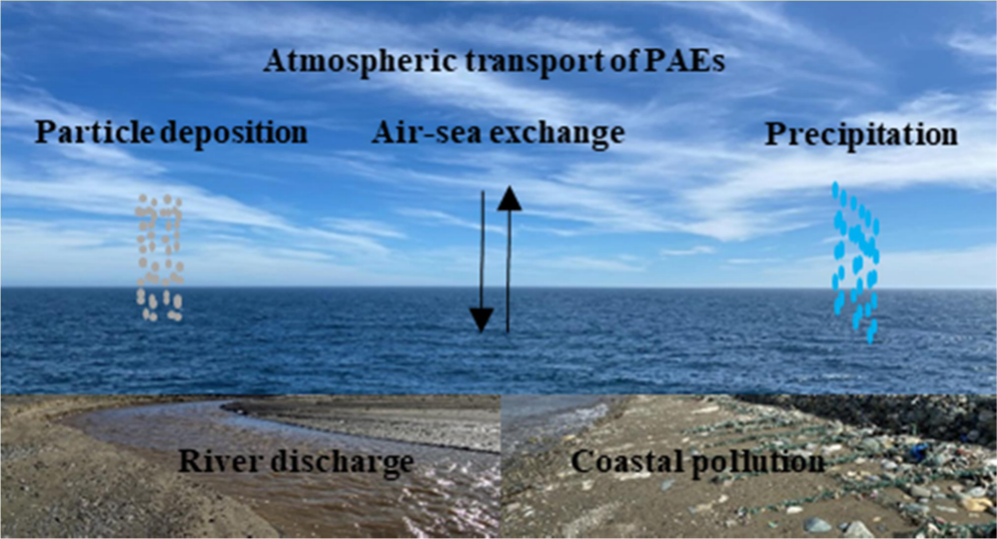

Phthalate esters (PAEs) have been investigated in paired air and seawater samples collected onboard the research vessel SONNE in the South China Sea in the summer of 2019. The concentrations of ∑_7_PAEs ranged from 2.84 to 24.3 ng/m^3^ with a mean of 9.67 ± 5.86 ng/m^3^ in air and from 0.96 to 8.35 ng/L with a mean of 3.05 ng/L in seawater. Net air-to-seawater deposition dominated air–sea exchange fluxes of DiBP, DnBP, DMP, and DEP, while strong water-to-air volatilization was estimated for bis(2-ethylhexyl) phthalate (DEHP). The estimated net atmospheric depositions were 3740 t/y for the sum of DMP, DEP, DiBP, and DnBP, but DEHP volatilized from seawater to air with an average of 900 t/y. The seasonally changing monsoon circulation, currents, and cyclones occurring in the Pacific can significantly influence the concentration of PAEs, and alter the direction and magnitude of air–sea exchange and particle deposition fluxes. Consequently, the dynamic air–sea exchange process may drive the transport of PAEs from marginal seas and estuaries toward remote marine environments, which can play an important role in the environmental transport and cycling of PAEs in the global ocean.

## Abstract 2

Phthalate esters are ubiquitous in the global environment and subject to atmospheric and oceanic transport to the South China Sea.

## Introduction

Phthalate esters (PAEs) are synthesized organic chemicals and have been used on a large scale as plasticizers and additives for more than 80 years. Owing to their versatility, PAEs are extensively used in the manufacturing of resin and polymer products, building materials, personal care products, medical devices, food and water packages, and pharmaceuticals, which account for more than 80% of worldwide plasticizer production.^1−3^ The annual global production of phthalates was 4.7 million tons in 2006 and increased to about 8 million tons in 2015.^4^ Previous studies have shown that phthalates are reproductive and developmental toxicants; hence, PAEs are considered endocrine-disrupting chemicals (EDCs) and pose a toxic risk to organisms.^5−7^ Six PAEs, e.g., dimethyl phthalate (DMP), diethyl phthalate (DEP), di-*n*-butyl phthalate (DnBP), butyl benzyl phthalate (BBP), bis(2-ethylhexyl) phthalate (DEHP), and di-*n*-octyl phthalate (DnOP), have been listed as priority control pollutants by the US Environmental Protection Agency (EPA), the European Union, and China.^4,8^

As PAEs are physically added to the products, they can leach out during production and application, especially from the disposal of plastic waste.^9^ Continuous emissions of PAEs have led to their ubiquitous distribution and abundance in the global environment. For example, PAEs have been determined in wastewater, river water, seawater, air, sediment, and fish.^10−15^ Both atmospheric and aquatic transport pathways play an important role in the presence of PAEs in the marine environment.^16−19^ When PAEs enter the marine environment, they can redistribute in different environmental compartments and exchange, crossing the interface between gas/particle, air/water, water/sediment, and water/organism.^19−21^ Furthermore, it has been proven that sediments are important sinks and sources for PAE distribution and bioaccumulation in the marine environment.^22−24^ Besides, photo- and biological degradation may interfere with their levels in the air and aquatic environments.^25,26^ However, the transformed products of PAEs can be more toxic than their parent substances.^27^ Consequently, air–sea exchange processes may significantly interfere with the biogeochemical cycle of PAEs in the marine environment.^1^

The South China Sea is one of the largest marginal seas in the world, which is surrounded by China and several countries in Southeast Asia within the tropical–subtropical climate realm. Previous studies have highlighted that riverine discharges are important vectors for various classes of organic contaminants in the South China Sea, such as from the Pearl River and the Mekong.^28,29^ Several classes of organic contaminants have been measured in seawater, air, sediments, and fish species, indicating that the South China Sea serves as a sink for emerging and legacy organic contaminants.^30−34^

To the best of our knowledge, there is no relevant report for PAEs offshore of the South China Sea. Hence, it is requested that the levels and environmental fates of PAEs in the South China Sea be thoroughly investigated so as to close the gap. In this work, we aim to (1) determine the concentrations, congener profiles, and spatial distributions of major PAEs in air and seawater obtained from a research ship campaign across the south-to-north transect in the South China Sea, (2) estimate air–sea exchange fluxes of PAEs, and (3) calculate atmospheric particle-bound PAE depositions into the South China Sea. This work provides a consistent unique dataset for evaluating the risk of PAEs to the ecosystem of the South China Sea.

## Materials and Methods

### Collection of Samples

Ship-bound sampling was carried out onboard the German research vessel SONNE (SO269) along a transect from Singapore to Hong Kong in 2.8-2.9.2019. Air and water samples were collected intensively offshore of the South China Sea (Figures S1 and S2). In summary, an actively high-volume air sampler was deployed at the Monkey deck (15 m above sea level) and ran at 15 m^3^/h for 24-h samples. A quartz fiber filter (QFFs, diameter: 150 mm, pore size: 1.0 μm) was used to trap particle-phase PAEs, and the gaseous PAEs were collected by a follow-up PUF/XAD-2 resin glass column. As reported by Lohmann et al.,^35^ there is always the potential for contamination by air from shipboard samples. Therefore, the air sampler was usually running continually in the headwind, with wind speed > 3 m/s. Sometimes, the pump was stopped when air masses came from the backside to avoid combustion emissions. Air sample blanks (column and filter) were prepared by briefly opening the PUF/XAD-2 column and filters next to the air sampler. Air samples were stored at 5 °C in the cooling room, and QFF filters were stored at −20 °C.

Seawater sampling was performed in the water lab via a seawater intake system, which is built with stainless steel tubing for the whole system; the inlet is located at 1 m underneath the keel (8 m depth). High-volume seawater samples were collected with a glass fiber filter (GFF, 1.2 μm, 140 mm) for the particles and an XAD-2 resin column for the dissolved PAEs. The volumes of seawater samples ranged from 36 to 310 L. All samples were transported with cooling containers from Hong Kong back to Helmholtz-Zentrum Hereon, Germany, in December 2019. Detailed information on air and water samples is provided as summaries in the Supporting Information (Tables S1 and S2).

### Sample Preparation and Instrument Analysis

The air and seawater samples were treated in a clean laboratory, as described in previous work.^1^ In brief, PUF-XAD-2 columns (vapor phase) and XAD-2 columns (dissolved phase) were spiked with internal standards (d4-DMP, d4-DEP, d4-DiBP, and d4-DEHP: 50 μL × 100 ng/mL, LGC, Wesel) and extracted using a modified Soxhlet system with dichloromethane (DCM, Promochem, Wesel) for 16 h. QFF and GFF samples were extracted in that way as well. The extracts were concentrated and cleaned up with column chromatography packed with 2.5 g of silica gel (Macherey Nagel, Düren, Germany) and 3 g of anhydrous sodium sulfate (99%, Merck, Darmstadt, Germany) on the top. The extracts were blown down to 190 μL under a gentle nitrogen stream (purity: 99.999%) and spiked with 10 μL of 50 pg/μL isotope-labeled poly(chlorinated biphenyl) 208 (^13^C_12_-PCB 208, Cambridge Isotope Laboratories) as an injection standard. The samples were determined for 7 PAEs, including DMP, DEP, DiBP, DnBP, BBP, DCHP, and DEHP, which are supplied by LGC (Wesel). The physicochemical properties of the PAEs are listed in Table S3, and the information on the chemicals and materials is given in Table S4.

Analysis was performed with a gas chromatograph (Agilent 8890A) coupled to a triple quadrupole mass spectrometer (Agilent 7010B, GC–MS/MS) equipped with a programmed temperature vaporizer (PTV) injector (Agilent Technologies). The MS transfer line and the high-sensitivity electron impact ionization source (HSEI) were held at 280 and 230 °C, respectively. The MS/MS was operated in multiple reaction monitoring (MRM) mode. Two HP-5MS columns (15 m × 0.25 mm i.d. 0.25 μm film thickness, J&W Scientific) were connected in tandem for separation. The details for the instrumental method and the quantifier ions are listed in Tables S5 and S6.

### Quality Assurance and Quality Control

It is very critical to determine trace concentrations of PAEs in environmental samples, as relatively high contamination of PAEs might be present in the sampling material, equipment, and the surrounding environment. To eliminate the PAE contamination, the sampling materials, e.g., PUF/XAD-2 and XAD-2 columns, were cleaned with methanol, acetone, and *n*-hexane, in turn for 72 h, and all organic solvents were distilled prior to use. QFFs and GFFs were, respectively, baked out at 600 and 450 °C. Besides, dust, fibers, and microplastics present in the laboratory air and the research vessel may cause significant PAE contamination during the sampling process.^36^ In this work, self-designed columns and samplers were applied for air and seawater sampling (Figure S3), which reduced the exposure time to indoor air, thus eliminating contamination onboard the research vessel and in the analytical laboratory.^37^ Moreover, an artificial aspect often occurs for particles with QFF or GFF for high-volume air and water sampling. Fine particles might penetrate the filters and be trapped by PUF or XAD-2, and the particle-bound PAEs will be extracted as a gaseous phase or dissolved phase, especially for high molecular PAEs. However, the analysis of the concentrations of PAEs in air and seawater is usually not impacted significantly.^37^ The effects of sampling volumes on the recoveries for air and seawater sampling were studied by field spiking of deuterated PAEs.^38^ The recoveries for sampling 500 L seawater were 48% for d4-DMP, 52% for d4-DEP, 88% for d4-DnBP, and 100% for d4-DEHP, respectively. The losses of d4-DMP and d4-DEP may result from their relatively high solubility in water, which suggests the concentrations of DMP and DEP in seawater might be underestimated. The recoveries of deuterated PAEs in spiked air samples ranged from 80 to 140%, suggesting that PUF/XAD-2 columns are efficient for sampling trace PAEs in air and losses of PAEs during storage are not significant. Besides, the recoveries of breakthrough tests showed that 80% PAEs retained on the first column, indicating that no significant breakthrough happens for PAEs.^38^

DMP, DEP, DiBP, DnBP, and DEHP were detectable in the field blank of PUF/XAD-2, XAD-2 columns, and QFF filters. The method detection limits (MDLs) were defined by the mean concentrations in blanks plus three standard deviations of the blanks. The MDLs ranged from 0.002 ng/m^3^ DCHP to 0.15 ng/m^3^ for DEHP in the gaseous phase, from 0.001 ng/m^3^ for DCHP to 0.072 ng/m^3^ for DEHP in the particle phase, from 0.001 ng/L for DCHP to 0.17 ng/L for DEHP in the dissolved water phase, and from 0.001 ng/L for DCHP to 0.47 ng/L for DEHP in GFF (particular matter) (Table S7). It is noted that the MDLs were calculated with an average volume of 370 m^3^ for PUF/XAD-2, 180 m^3^ for the particle phase, and 170 L for seawater. The concentrations of PAEs reported in this work were not corrected with their blanks.

### Air Mass Back Trajectory

The air mass origins of the air samples were assessed using air mass back trajectories (BT), calculated by the Hybrid Single-Particle Lagrangian Integrated Trajectory model (HYSPLIT).^39^ Air mass back trajectories traced back the air masses for 120 h with 6 h steps at the heights of 10 m above the sea level, which are shown in Figure S4.

### Data Analysis

The data of GC–MS/MS were analyzed using MassHunter B10 (Agilent). Statistical analyses were performed with Excel 2016 and Origin 2020 (OriginLab). Geographic maps were plotted with Ocean Data View 4.0.^40^ The washout ratio, air–sea exchange, and atmospheric particle deposition were calculated on the basis of the equations previously reported.^1,19^ The details of the calculation methods are presented in the Supporting Information.

## Results and Discussion

### PAEs in Air

Seven PAEs were detected in gaseous and particle phases of ship-bound air samples collected in the South China Sea, which are summarized in Table S8 in detail. The concentrations of ∑_7_PAEs varied between 2.83 and 58.9 ng/m^3^, with a mean of 11.6 ± 11.3 ng/m^3^. DiBP, DnBP, and DEHP were the predominant PAEs, with average concentrations of 5.18 ± 5.92, 3.94 ± 3.62, and 1.47 ± 1.37 ng/m^3^ respectively, accounting for 89.2% of the ∑_7_PAEs. Other PAEs, such as DMP (mean: 0.74 ± 0.95 ng/m^3^), DEP (mean: 0.23 ± 0.14 ng/m^3^), BBP (mean: 0.04 ± 0.02 ng/m^3^), and DCEP (mean: 0.01± 0.01 ng/m^3^) showed relatively low levels in air. As compared with recent studies for PAEs in air, the concentrations of PAEs in air samples from this work were slightly lower than those measured in fine particulates at Yongxing Island (∑_5_PAEs: 3.8–160 ng/m^3^, mean: 16.6 ng/m^3^),^41^ 1–2 orders of magnitude lower than those measured in the urban areas,^42,43^ comparable to the levels, in the Asan Lake of Korea (∑_14_PAEs: 3.92–33.09 ng/m^3^)^44^ and the Chao Lake of China (∑_6_PAEs: 2.0–14.8 ng/m^3^),^45^ and higher than those reported for the North Sea (∑_6_PAEs: 2.57–7.82 ng/m^3^)^19^ and the Arctic (∑_6_PAEs: 1.11–3.34 ng/m^3^).^1^ The composition profile of PAEs in gaseous and particle phases varied in comparison with those from the urban sites; for instance, low proportions of DMP and DEP were found in air, although they are very volatile. We suppose the distribution pattern of PAEs in air could be caused by the origin of air masses, metrological conditions, and physicochemical behavior of PAEs, which are discussed in the context.

### Spatial Distribution of PAEs in Air

**Figure 1:**
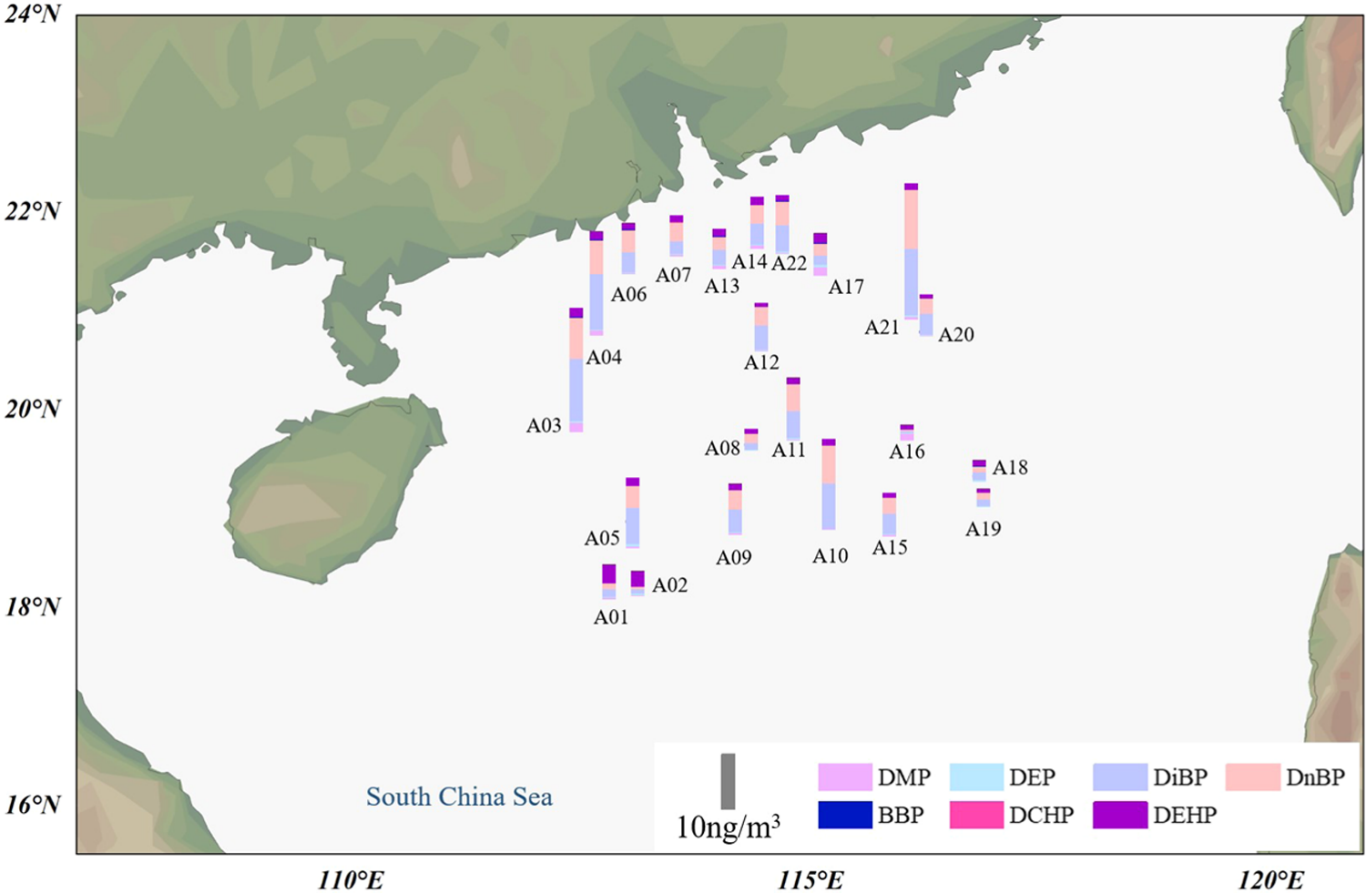
Spatial distribution of PAEs in the atmosphere (ng/m^3^) over the South China Sea. DiBP and DnBP are the predominant PAEs in air samples from A3 to A22, while DEHP prevails in air samples A1 and A2.

The spatial distribution of PAEs in the air of the South China Sea is shown in Figure 1, and the air mass back trajectories for each air sample are presented in Figure S4. The highest concentration of ∑_7_PAEs (24.3 ng/m^3^) was present in the air sample A21, which is almost in the range of PAE concentrations measured in urban environments.^42,43^ The BTs (Figure S4) show that 50% of air masses are from the coastal region of Vietnam, other 31% are from southwestern Asia, and 19% are from the East China Sea. Typically for the summer season, the trade wind transports the air masses mainly over South Asian countries such as Malaysia, Singapore, and Indonesia. Other higher concentrations were presented in air samples collected along the Chinese coast, such as A3 (22.1 ng/m^3^), A4 (18.5 ng/m^3^), and A10 (16.1 ng/m^3^). BTs showed that important sources of air masses were from the Chinese coastal areas of Fujian and Guangdong provinces, with contributions of 55 and 71% for A3 and A4. Another major air mass source was in Southeast Asia; it swept through Vietnam and its east coast region (Figure S4). Relatively low PAE concentrations were measured in air samples from offshore (A16, A18, A19), which are influenced by the summer monsoon with the air masses mainly from the open South China Sea, the Indian Ocean, and the Pacific Ocean. Besides, the atmospheric concentrations of PAEs measured in this work have also shown the impacts of weather conditions in the South China Sea. For instance, the rainfall precipitation could significantly deplete the PAE concentrations in air, which led to low levels in A1 and A2. Furthermore, several tropical cyclones, e.g., Lekima, Krosa, Bailu, and Podul, were formed in the North Pacific Ocean in August 2019 and moved northward during the sampling campaign.^46^ The tropical air masses and rainfall caused by the cyclones strongly influenced the South China Sea and Southeast China. Thus, relatively low concentrations of PAEs were measured in nearshore air samples A6, A7, A13, and A14. Nevertheless, the origin of the air masses and weather conditions can significantly affect the PAE concentrations in the South China Sea.

### Gas/Particle Partitioning

PAEs have been determined separately in gaseous and particle phases that allow evaluation of the gas/particle processes (Table S9). The particle-bound fractions (ø) showed that DMP (0.06 ± 0.04) and DEP (0.32 ± 0.19) were predominantly in the gas phase, whereas DiBP (0.47 ± 0.37), DnBP (0.49 ± 0.35), and DEHP (0.48 ± 0.16) were more present in the particle phase (Table S9). In comparison to the particle-bound fraction values calculated in other regions, DMP is quite comparable to the city of Paris (DMP: 0.07), but DEP, DnBP, and DEHP are much higher (DEP: 0.06, DnBP: 0.13, DEHP: 0.35).^47^ The fraction values of DnBP and DEHP are similar to those determined in the Gulf of Mexico (0.32 for DnBP and 0.43 for DEHP).^48^ In the North Atlantic and the Arctic, the particle-associated fraction of DnBP (0.46) is similar to this study, but DEHP (0.78) is much higher than in this work.^1^

Indirect photolysis in air, caused by hydroxyl radical attack in both gaseous phase and particle-bound phthalates, may play a significant role in the removal of PAEs. The estimated half-lives of PAEs were 0.38, 0.75, 0.89, 2.39, and 14.41 days for DEHP, BBP, DnBP, DEP, and DMP,^49^ suggesting that DEHP, BBP, and DnBP should be degraded more rapidly during atmospheric transport and that DEP and DMP are rather persistent during atmospheric transport. As atmospheric reactions often deplete compounds in the gaseous phase, the partitioning of high molecular weight PAEs to particles, e.g., DEHP, might reduce their photolysis rates and thereby increase their persistence in the atmosphere.

### Washout Ratio

The washout ratio (WR) estimated is 240,000 ± 9900 for DMP, 75,200 ± 15,800 for DEP, 19,300 ± 510 for DiBP, 19,300 ± 480 for DnBP, and 9780 ± 3260 for DEHP (Table S10), showing that DMP and DEP can be easily scavenged from air than other PAEs. DEHP has relatively low W*R* values, suggesting they are more persistent in the atmosphere. These findings are proved by varying PAE profiles presented in air samples A1 and A2, which were collected when it was raining. The concentrations of DMP, DiBP, and DnBP in A1 and A2 decreased by a factor of 2–5 in comparison to A3 and A4. Given the high intensity of rainfall in the summer, precipitation scavenging is a significant process to remove DMP and DEP from the atmosphere in the South China Sea, which is consistent with the model prediction for wet deposition of other semivolatile organic compounds in the tropic ocean.^50^ Compared with the WR values reported for DnBP and DEHP in the North Sea, the variations in WR are highly influenced by both environmental temperatures and particle-bound fractions. Although DMP and DEP are relatively volatile and resistant to photodegradation in air, their high washout ratios suggest that they are limited for long-range atmospheric transport (LRAT) and turn to deposit in the water column. While DiBP, DnBP, and DEHP can undergo medium LRAT, and their particle-bound fractions might be favored for LRAT.

### PAEs in Seawater

**Figure 2:**
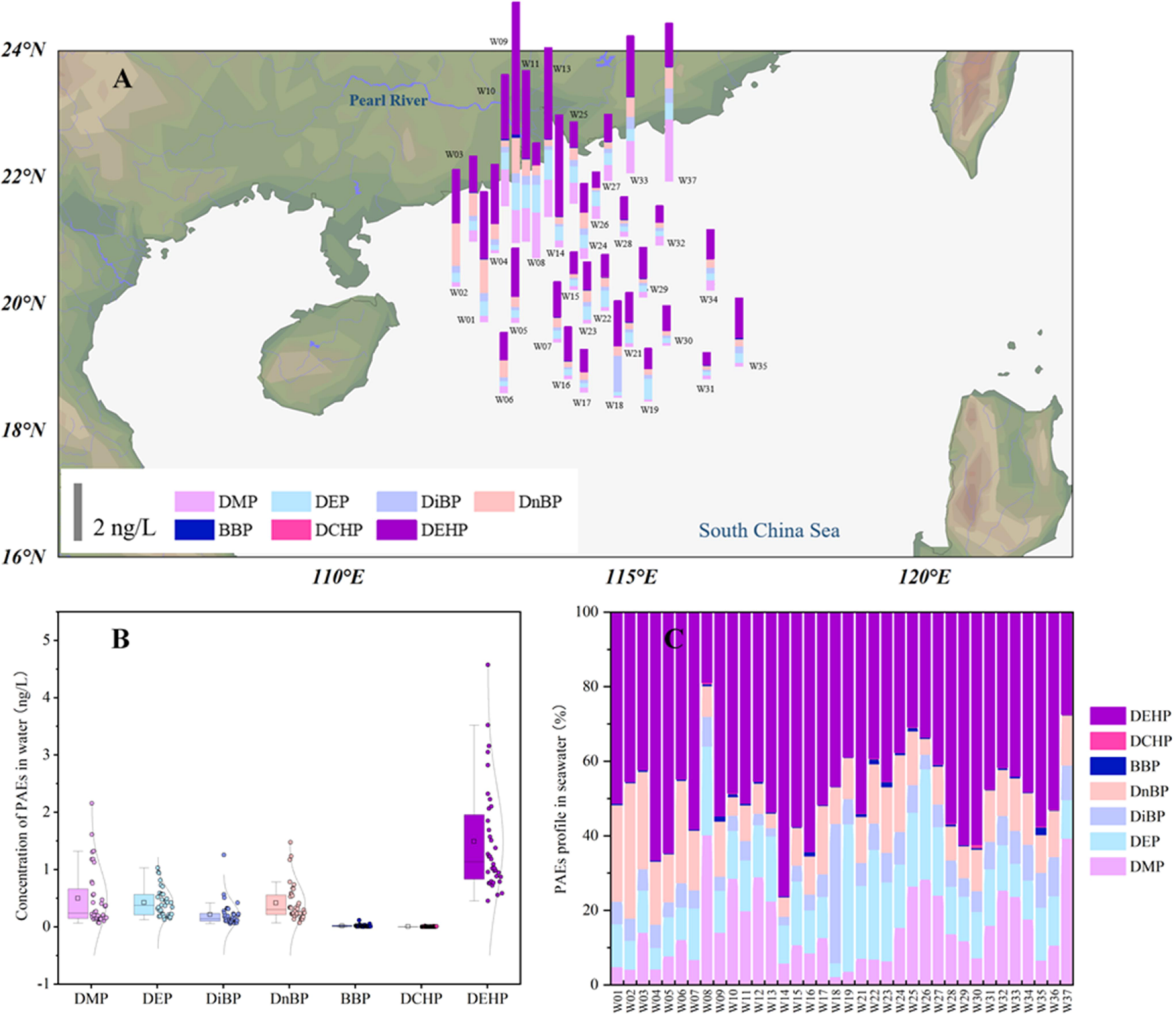
(A) Spatial distribution of PAEs in seawater of the South China Sea; (B) concentration ranges of individual PAEs (ng/L); and (C) profile of PAEs in individual water samples.

The concentrations of PAEs in seawater in the South China Sea are shown in Figure 2, and detailed information is summarized in Table S11. The concentrations of the ∑_7_PAE in seawater ranged from 1.08 to 9.82 ng/L with a mean of 3.48 ± 1.89 ng/L. DEHP was the dominant PAE in seawater with a mean concentration of 1.79 ± 1.11 ng/L, followed by DMP (0.51 ± 0.52 ng/L), DEP (0.46 ± 0.26 ng/L), DnBP (0.46 ± 0.36 ng/L), and DiBP (0.24 ± 0.22 ng/L). Concentrations of BBP and DCHP are only detectable in a few seawater samples with a mean concentration of 0.020 ± 0.021 and 0.003 ± 0.003 ng/L, respectively. Given the high water solubility of DMP (5220 mg/L) and DEP (591 mg/L), breakthrough might occur for large-volume seawater samples; thus, the concentrations of DMP and DEP might be underestimated on account of limitations of the high-volume sample collection.^19^

The PAE concentrations clearly showed spatial distribution, which is higher near the coast, and decline from nearshore to offshore. The high levels were presented in the water plume of the Pearl River (Figure S5). Recently, PAEs were measured in the Pearl River, with ∑_6_PAEs (DMP, DEP, BBP, DnBP, DEHP, DOP) ranging from 500 to 28,100 ng/L, which are 2–3 orders of magnitude higher than this study.^51^ Evidently, the river discharge of the Pearl River could be a major input vector for PAEs in the Pearl River Delta and the South China Sea.^52^ Compared with other coastal regions of East Asia (Table S12), the PAE concentrations are 1–3 orders of magnitude lower than those in the Bohai Sea and the Yellow Sea (∑_16_PAEs: 2880 and 1600 ng/L),^53^ in the Yangtze River Estuary (∑_16_PAEs: 180–3421 ng/L),^18^ and in the East China Sea and Korean South Sea (∑_7_PAEs (DMP, DEP, DiBP, DnBP, BBP, DEHP, DOP): 63–169 ng/L).^23^ In comparison with global oceans (Table S12), the PAE concentrations in the South China Sea are also at lower levels. They are 2–3 orders of magnitudes lower than those from Pradu Bay, Thailand,^54^ Chabahar Bay, Iran,^55^ and Marseille Bay (NW Mediterranean Sea)^56^ and in the surface microlayer and sea surface water in the Antarctic (77–406 ng/L). While the concentrations of PAEs are about 5–10 times lower than those reported from the Pacific Ocean,^57^ and in good agreement with those measured in the North Sea,^19^ the North Atlantic, and the Arctic (0.03–5.03 ng/L).^1^ Oceanic current dilution (Figure S5), as well as enhanced photodegradation and biodegradation under the tropical climate, may be responsible for the large differences in concentration between the open South China Sea and other coastal areas.^4,23^

### Water-Suspended Particular Matter Partitioning

PAEs have also been detected in suspended particular matters (SPMs) in seawater. The detection frequencies of PAEs in SPMs were 97% for DnBP, followed by BBP (75%), DEP (69%), DMP (53%), and DEHP (47%), while only 8% for DiBP and DCHP (Table S11). As shown in Table S13, SPM-bound PAE fractions were 0.34 ± 0.16 for DEHP, followed by 0.25 ± 0.14 for DiBP, 0.23 ± 0.14 for DnBP, 0.19 ± 0.09 for DEP, and 0.08 ± 0.07 for DMP. Compared with other studies, the TSM fraction of DEHP is in line with the values determined in the North Sea (0.42),^19^ whereas it is lower than those determined in the Lake Yssel and the Rhine (0.67).^58^ However, the TSM fractions of DnBP and DiBP are higher than the North Sea (0.02) and the Rhine (0.02) but similar to the fractions (0.14–0.34) measured in the River Mersey Estuary in the U.K.^59^ The differences might be caused by the seawater sampling technique, the character of different SPMs, and water chemistry. The elevated seawater temperature in the tropical ocean usually lowers the SPM fractions of PAEs. However, the competition between absorption and degradation may lead to complicated water–SPM partitioning. Nevertheless, SPM-bound PAEs are expected to be accumulated in the sediment and undergo slow degradation processes.^60^

### Environmental Source of PAEs

PAEs in the South China Sea are usually transported from terrestrial sources, such as volatilization during the production of plastic products, e-waste treatment, and riverine input from surrounding countries, which have been evidenced by the high PAE levels measured in indoor air, urban air, and adjacent rivers.^52,61−63^ For instance, the sum concentrations of DMP, DEP, DnBP, and DEHP were measured in fine atmospheric particles (PM_2.5_) of metropolitan areas of Guangzhou (32.5–76.1 ng/m^3^), Shanghai (10.1–101 ng/m^3^), Beijing (8.02–107 ng/m^3^), and Harbin (13.5–622 ng/m^3^),^43^ and 13 PAEs (range: 2.6–15.3 ng/m^3^) were measured in atmospheric particles over the inland lake Chaohu.^45^ Fang et al. reported DnBP and DEHP in atmospheric particles from various sites in central Taiwan, with respective mean concentrations of 33.94 and 109.7 ng/m.^3,42^ These concentrations of PAEs over land or islands are usually 1–2 orders of magnitude higher than those reported in the South China Sea.

The river-run discharge from the Pearl River is an important vector for the occurrences of PAEs in the South China Sea.^64,65^ In addition, PAE contaminations accumulated in neighboring marginal seas could also be transported into the South China Sea by the Chinese coastal current.^66^ For instance, PAEs discharged from the Yangtze River are an important source for the East China Sea, which can be further transported to the South China Sea via Taiwan Strait. Besides, the Kuroshio Current has important effects on both physical and biological processes of the North Pacific, including nutrient, sediment, and pollution transport.^67,68^ Hence, PAEs discharged into the coastal areas could be partially transported to the South China Sea with the ocean currents. During the sampling cruise, the basin-wide circulation of the South China Sea was influenced by the South and East Asian summer monsoons in August,^69^ and several cyclones occurred in the western Pacific Ocean. The cyclonic gyres significantly accelerate the matter exchange between the continental runoff and the Pacific oceanic water masses, which led to a strong dilution of the PAE concentrations observed in this study.

In addition, terrestrial wastes and matters, e.g., plastic litter or microplastics, could be transported to the South China Sea and release PAEs into air and seawater.^70−72^ It is estimated that up to 300,000 t/y of plastic are discharged from the surrounding rivers into the South China Sea,^73^ and as many as 2.56–7.08 million tons of plastic pollution to the ocean every year from the countries bordering the South China Sea.^70^ Besides, about 1400 t microplastics entered the South China Sea by means of dry deposition.^74^ As PAEs are the major plasticizers physically added to plastic matter, they can leach out from plastic materials and release them into the water columns. DiBP and DnBP are the two main PAEs released from polyethylene bags, while DMP and DEP are mainly discharged from poly(vinyl chloride) products.^75^ Microplastics in the ocean could efficiently release plastic additives, including PAEs, while the leaching process could be slowed down when microplastics are covered by a biofilm or buried in deep-sea sediment.^76^

### Air–Sea Exchange

**Figure 3:**
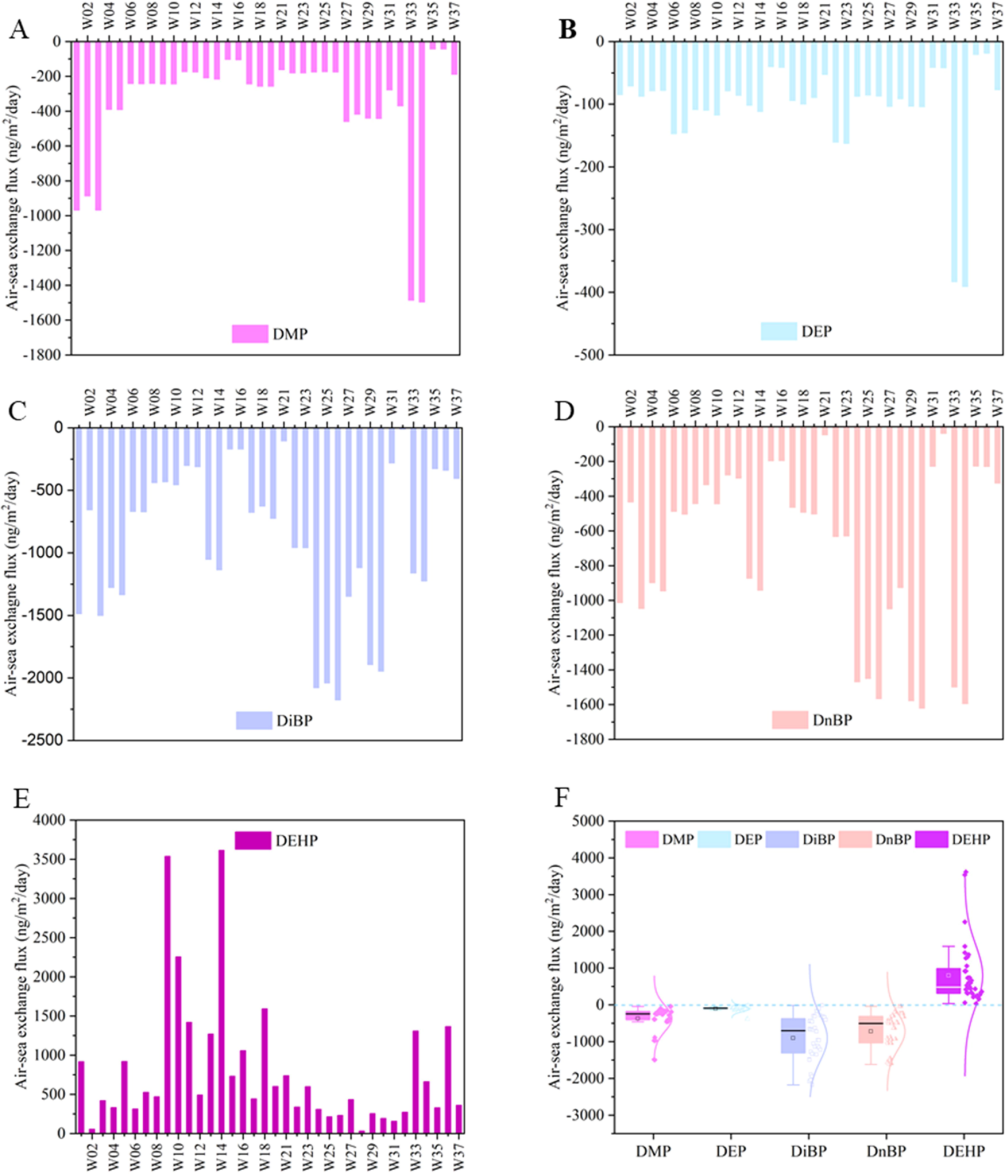
Air–sea exchange fluxes of PAEs (ng/m^2^/day) are calculated with two-film fugacity mode using paired air/seawater concentrations. The positive (+) value indicates water-to-air volatilization, and the negative (−) value means air-to-water deposition. (A) DMP, (B) DEP, (C) DiBP, (D) DnBP, (E) DEHP, and (F) the range of the air–sea exchange fluxes of individual PAEs.

The estimated air–sea exchange fluxes of PAEs in the South China Sea are shown in Figure 3; the details are summarized in Table S16. BBP and DCHP are excluded due to their low detection frequencies and low concentrations. Net volatilization dominated the air–sea exchange fluxes of DEHP, with an average of +800 ± 360 ng/m^2^/day, which is about 5–20 times higher than those estimated for the North Sea (+53 ± 24 ng/m^2^/day)^19^ and for seawater off the Norwegian coast (+212 ng/m^2^/day),^1^ while it changed to air-to-water deposition in the open ocean and the high Arctic.^1^ In the South China Sea, the higher net volatilization fluxes of DEHP were estimated for the water/air sample pairs W9/A9, W14/A10, and W10/A9, which are consistent with the high concentrations of dissolved DEHP in seawater from the offshore. Although the lower molecular weight PAEs are favored to partition to the gaseous phase, once they deposit into seawater, their high solubility in water and very low *K*_AW_ values limit their volatilization ability from the aquatic phase to air.^77^ On the other hand, the air–water partition coefficients (*K*_AW_) increase with growing molecular weight; hence, PAEs with higher molecular weight, such as DEHP, can potentially vaporize more rapidly from water to air, which aligns with the air–sea exchange fluxes of DEHP estimated in this work and other existing literature.^1,19^ These findings suggest that PAEs are favored for both atmospheric and oceanic transport. Nevertheless, the air–sea exchange of PAEs occurring at the interface may drive the transport of PAEs from the marginal seas to remote oceans following the mode of “grass hopping”.^78^

Though the concentrations of PAEs in the gaseous and dissolved phases and the physiochemical behaviors determine the direction and intensity of the air–sea exchange, several other important factors can affect the air–sea exchange fluxes of PAEs. Air masses circling in the South China Sea during the sampling period are dominated by summer monsoons. The clean oceanic air may dilute the PAEs emissions from terrigenous origins and decrease the air concentration gradients for the entire South China Sea. Consequently, the potential for water-to-air volatilization can be increased. Winter monsoons might intensify air-to-water deposition because the air masses originating from East Asia can significantly transport land emissions of PAEs to the South China Sea. Besides, the loss of PAEs through the sinking of sediment might cause a decline of dissolved PAE concentrations in seawater and lead to the tendency of air–water exchange of PAEs from air to water, especially for high molecular weight PAEs. Furthermore, microbial degradation and adsorption of PAEs by phytoplankton in seawater could also eliminate dissolved PAE concentrations,^75^ thereby increasing the air-to-water deposition potential. Other meteorological parameters, such as both temperature and wind speed, can also influence the air–water exchange fluxes by altering the total mass transfer coefficient.^79^ For instance, the net volatilization fluxes of DEHP were estimated at 57 ng/m^2^/day for water sample W2 (wind speed: 1.1 m/s) and 33 ng/m^2^/day for W28 (wind speed: 1.5 m/s), which are 20 times lower than the average of 800 ng/m^2^/day. This fits previous studies for PCBs in the South China Sea and the Pacific Ocean.^80^

### Atmospheric Particle Deposition

**Figure 4:**
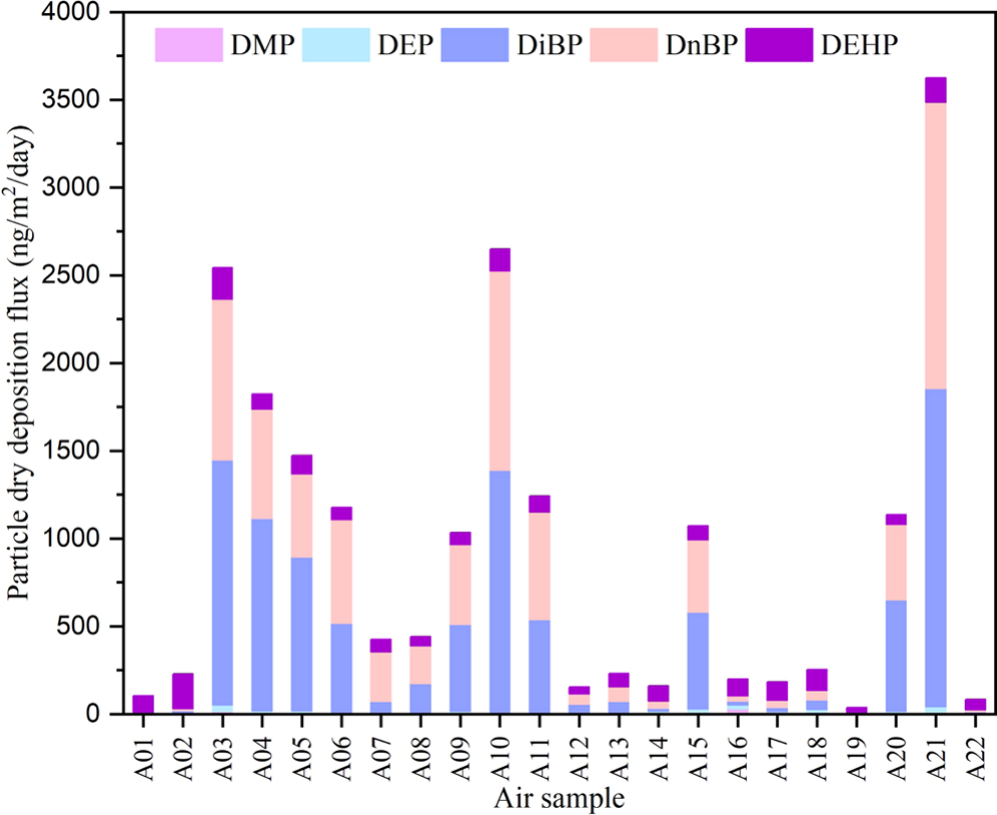
Dry deposition fluxes of particle-bound PAEs (ng/m^2^/day) to the South China Sea, which were dominated by DiBP and DnBP.

As shown in Figure 4, the dry deposition fluxes of total PAEs ranged from 34 to 3620 ng/m^2^/day with an average of 920 ± 990 ng/m^2^/day (Table S17). The dry deposition fluxes of individual PAEs were dominated by DiBP (440 ± 550 ng/m^2^/day), DnBP (370 ± 430 ng/m^2^/day), and DEHP (91 ± 42 ng/m^2^/day), which is consistent with the composition profile of PAEs in the particle phase. The highest dry deposition fluxes were determined near the coastal area of Southeast Asia, followed by several higher values along the coastal area of Southeast China. The lower dry deposition fluxes were estimated for air samples collected during rainfall since precipitation has significantly decreased the particle dry deposition. In addition, atmospheric circulation and continental or oceanic air masses are considered the main factors that influence the dry deposition fluxes of PAEs. The air masses off inland may bring high particle load into the coastal region, thereby increasing the dry deposition of PAEs in the marine environment. As the continental air masses prevail in fall and winter over the study area, it is suggested that dry deposition may play an important role in winter months in the South China Sea. In comparison with other classic organic contaminants, particle deposition fluxes of PAEs are less than that of decabromodiphenyl ether (BDE-209) of 1670 ± 1940 ng/m^2^/day in the Pearl River Delta,^81^ 3 times higher than that of polycyclic aromatic hydrocarbon (PAH) of 260 ± 190 ng/m^2^/day observed near coast of the South China Sea,^82^ and 2 orders of magnitude higher than those of organochlorine pesticides (OCPs).^81^

### Atmospheric Dry Flux

**Figure 5:**
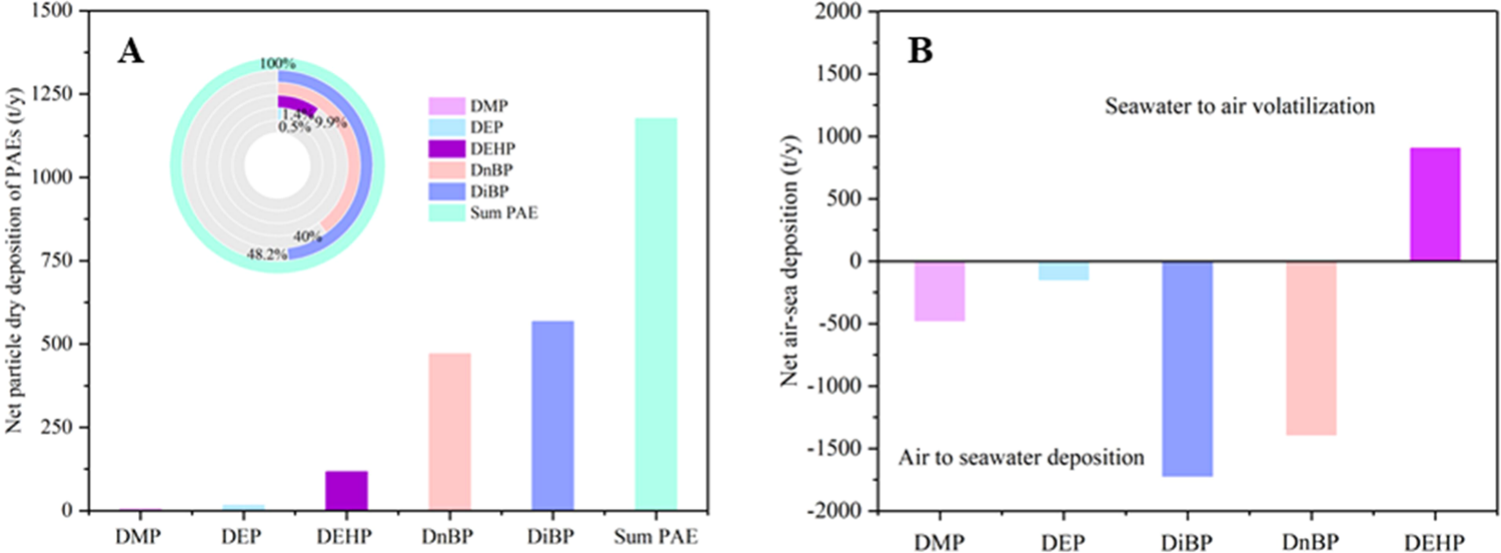
(A) Annual net dry deposition of particle-bound PAEs (t/y) and (B) the net air–sea exchange fluxes in the South China Sea (t/y). It should be noted that DEHP and DCHP showed net volatilization from water to air.

Given a surface area of approximately 3,500,000 km^2^ for the South China Sea,^83^ the atmospheric particle deposition fluxes of ∑_5_PAEs ranged from 46 to 4630 t/y, with a mean of 1180 ± 1260 t/y (Figure 5A). DiBP, DnBP, and DEHP accounted for 48.1, 40.0, and 9.9% of the total particle deposition of PAEs, respectively. Once deposited in the sea, PAEs may repartition between dissolved and particle phases in the water column and sink to the bottom of the ocean.^21^ PAEs have been measured in the sediment of the central Indian Ocean with concentrations ranging from 823 to 1615 ng/g dw.^84^ The atmospheric particle deposition can be an important source of PAEs in the oceanic sediment.^84^ Nevertheless, the occurrences and inventories of PAEs in the sediment of the South China Sea request further research.

Based on the air–sea exchange fluxes of PAEs, the net depositions were dominated by DiBP and DnBP as well, with an average of 1150 and 920 t/y, followed by 470 t/y for DMP and 140 t/y for DEP (Figure 5B). The net volatilization of DEHP was estimated to be 1020 t/y from the South China Sea, which can be transported further to remote oceans via the atmosphere. The air–sea exchange fluxes of PAEs in this work were significantly higher than those estimated for the North Sea and the European Arctic.^1,19^ The overall net fluxes are 3740 t/y deposition for DMP, DEP, DiBP, and DnBP, and 900 t/y volatilization for DEHP in the South China Sea.

## Implications

This study showed the dynamic air–sea exchange process may drive the transport of PAEs from contaminated marginal seas and estuaries toward remote marine environments, which can play an important role in the environmental transport and cycling of PAEs in the global ocean. The changing meteorological conditions, especially through the seasonality of the monsoon, have a strong impact on the variability of the air–sea exchange. So far, only a few regulations have been implemented to limit the applications of PAEs. Indeed, plastic debris or microplastics are a kind of intermediate storage and transport media for PAEs from sources to remote areas, and these release them into the local environment during life. On the other hand, the most toxic risk of microplastics to marine organisms can be attributed to the chemical additives such as PAEs, softeners, antioxidants, and flame-retardants. Therefore, research on the interaction of microplastics and synthetic organic plastic additives in the marine environment including the interface interaction, sedimentation, and bioaccumulation in the organisms needs to be strengthened in future studies.

## Supporting Information Available

Additional supporting information, including the description of the methods for estimating air–sea exchange, atmospheric particle deposition, washout ratio, chemicals and reagents, the ODV maps for air and water sampling, air mass back trajectories, flow field of the South China Sea in August 2019, detailed information of air and seawater samples, analytical method of GC–MS–MS, concentrations of PAEs in all air and seawater samples, and estimated air–sea exchange fluxes and particle-bound OPE deposition fluxes (PDF)
