# Supplementary material for: Air–Sea Exchange and Atmospheric Deposition of Phthalate Esters in the South China Sea
Source: Environ Sci Technol. 2023 Jul 17;57(30):11195–205. doi: 10.1021/acs.est.2c09426 (PMC10399291; doi:10.1021/acs.est.2c09426)
Supplement: Supplementary file 1 — es2c09426_si_001.pdf [file es2c09426_si_001.pdf]

Supporting information

Air-sea exchange and atmospheric deposition of phthalate esters in the South China Sea

Lijie Mi[†,‡], Zhiyong Xie[†]*, Weihai Xu[□], Joanna J. Waniek[ǂ], Thomas Pohlmann[‡], Wenying Mi[□]

[†]Institute of Coastal Environmental Chemistry, Helmholtz-Zentrum Hereon, Geesthacht 21502, Germany

[‡]Institute of Oceanography, University of Hamburg, 20146 Hamburg, Germany

[□]Key Laboratory of Ocean and Marginal Sea Geology, South China Sea Institute of Oceanology, Chinese Academy of Sciences, Guangzhou 510301, China

[ǂ]Department of Marine Chemistry, Leibniz Institute for Baltic Sea Research Warnemünde, Rostock 18119, Germany

[□]MINJIE Institute of Environmental Science and Health Research, Geesthacht 21502, Germany

*Corresponding author: e-mail: Zhiyong.xie@hereon.de, Tel: +49 4152 872330

Pages: 39

Test: S1-S3

Figure S1-S5

Table S1-S17

Content

Text S1. Chemicals and materials

Text S2. Washout ratio

Text S3. Air-sea exchange

Text S4. Atmospheric particle deposition

Text S5. Pearson correlation analysis

Figure S1**.** Air sampling stations in the South China Sea, with the blue dots marked at the starting position of each air sample

Figure S2. (A) Seawater sampling stations in the South China, with the blue dots marked at the starting position of each air sample. The color map shows the salinity profile in surface seawater; (B) general water mass circulation in the research area in summer

Figure S3A. High-volume air sampler is equipped with quartz fiber filter for atmospheric particles and a PUF/XAD-2 column for gaseous phase. The air sampler is placed at the upper deck and operated with headwind. The Filter and PUF-XAD-2 column are changed at sampling site. The PUF/XAD-2 column can be sealed with glass caps immediately after its disconnection from the sampler to eliminate exposure time to the indoor air. The Quartz fiber filter is wrapped in clean aluminum. The PUF/XAD2 columns and filters are stored at -20°C; S3B: The set-up of the high-volume water sampler equipped with glass fiber filter and a XAD-2 column. The high-volume water sampler is connected to the ship-intake system with metal tubing. A glass fiber filter is closed in the filter holder to collect suspended particular matters and a XAD-2 column to retain organic chemicals in the dissolved phase. The seawater flows over the entire system at a flow rate of 0.2-1 L/min. The XAD-2 column is sealed with clips immediately after the sampling, and stored at 5°C. Glass fiber filter is wrapped in a clean aluminum foil and stored at -20°C.

Figure S4. Air mass back trajectories of air samples. Colors indicate the origin of the air mass parcels

Figure S5. Figure S4. The schematic diagrams of the flow field of the South China Sea in 7th (A) 13th (B), 19th (C) and 25th (D) in August 2019

Table S1. Detail information for air sampling

Table S2. Detail information for seawater sampling

Table S3. Information about chemicals in this study

Table S4. Physiochemical properties of seven phthalate esters

Table S5. Table S 5. Agilent 7890 GC-MS/MS conditions

Table S6. Detail information for the determination of PAEs using GC-MS/MS

Table S7. The field blanks and the method detection limits of PAEs in PUF/XAD-2, XAD-2 columns, QFF and GFF filters

Table S8. Concentrations of PAEs in the atmosphere over the South China Sea

Table S9. Particle-bound fractions (ø) of PAEs in air samples from the South China Sea. The particle-bound fractions are quite uncertain because BBP and DCHP were below MDLs in most particle samples.

Table S10. Washout ratio (W) calculated with particle bound fraction following eq. $W = (1-ø)(RT/H)+øW_p$

Table S11. Concentrations of PAEs in the seawater from the South China Sea

Table S12. Comparison with previous studies for the concentrations of seven PAEs in seawater (ng/L)

Table S13. Particle-bound fractions (ø) of PAEs in seawater from the South China Sea

Table S 14. Correlation analysis of PAEs in air

Table S 15. Correlation analysis of PAEs in seawater

Table S16. Air-sea exchange fluxes of PAEs in the South China Sea. Positive value indicates water to air volatilization and negative value indicates air to water deposition

Table S17. Particle dry deposition of PAEs in the South China Sea

Text S1. Chemicals and materials

PAEs standard mixture, containing Dimethyl phthalate (DMP), Diethyl phthalate (DEP), Diisobutyl phthalate (DiBP), Di-*n*-butyl phthalate (D*n*BP), Butylbenzyl phthalate (BBP), Dicyclohexyl phthalate (DCHP) and Di(2-ethylhexyl) phthalate (DEHP) at 1000 μg/mL each, and surrogate standards, e.g. deuterated dimethyl phthalate (DMP d4), diethyl phthalate (DEP d4), n-dibutyl phthalate (DnBP d4) and di(2-ethylhexyl) phthalate (DEHP d4), were supplied by LGC (Wesel, Germany).

Picograde® solvents, e.g. methanol, acetone, n-hexane and dichloromethane (DCM) were purchased from Promochem (Germany). Neutral silica gel (0.063-0.2 mm) was supplied by Macherey Nagel (Düren, Germany) and anhydrous sodium sulphate (99%) and XAD-2 resin was obtained from Merck (Darmstadt, Germany).

Text S2. Washout ratio

Considering both vapor and particle scavenging mechanism, washout ratio (W) can be expressed as equation 1,

$$W = (1-ø)(RT/H)+øWp \qquad (1)$$

where ø is the particle-bound fraction of the chemical, RT/H is the dimensionless Henry's law constant (H) at the ambient temperature (298 K), and WP is the particle scavenging coefficient[1]. A representative WP value of 20,000 is used for the calculation in this work.[2]

Text S3. Air-sea exchange

During the transport from continental sources to the South China Sea, PAEs may undergo several processes at the interface between air and seawater. The direction and fluxes of air-sea exchange of PAEs are mainly controlled by their physicochemical parameters such as Henry's Law Constants and the concentrations in air and seawater, and are influenced by metrological conditions. In this work, air-sea exchange fluxes of PAEs were calculated using the modified version of Whitman's two-film fugacity model, which has been extensively

applied for the evaluation of PAHs, PCBs and PAEs in the marine environment,[3-5] The volatilization and deposition fluxes (F_{vol} and F_{dep}, ng/m^2/day) are calculated as in equation 3 and 4,

$$K_{OL} = \left(\frac{1}{K_w} + \frac{1}{K_a * H}\right)^{-1} \quad (2)$$

$$F_{vol} = K_{OL} C_w \quad (3)$$

$$F_{dep} = K_{OL} C_a / H' \quad (4)$$

The net diffusive gas exchange flux (F, ng/m^2/day) is then calculated by subtracting the volatilization flux from the deposition flux, which is described as equation 5,

$$F = K_{OL}(C_w - \frac{C_a}{H'}) \quad (5)$$

where Cw (ng/m^3) and Ca (ng/m^3) are the dissolved- and gas-phase concentrations, (Cw-Ca/H´) describes the concentration gradient (ng/m^3), H´ is the dimensionless Henry's law constant. The K_{OL} (m/s) is the overall mass transfer coefficient, which contains contributions from the mass transfer coefficients of the water phase (kw) and the air phase (ka). The calculation of Kw and Ka for PAEs refer to the equations recommended by Schwarzenbach et al.[6] and Wanninkoff's quadratic relationship.[7] H´ of PAEs are corrected with temperature of seawater (Tw, K) and averaged salt concentration (Cs, 0.5 mol/L). The total propagation error in F was 45%, which is derived from an error propagation analysis from previous studies.[5] A positive F value indicates seawater to air volatilization, while a negative F means that deposition is dominating air-sea exchange flux.

Text S4. Atmospheric particle deposition

Atmospheric deposition is one of the key processes that remove pollutants from air and is considered the major source of organic matter and chemical pollutants into the marine environment. Based on the concentrations of

PAEs measured in the particle phase, the particle-bound dry deposition fluxes (F_D, ng/m^2/day) were calculated via equation (6)[6]:

$$F_D = C_p \times V_d \quad (6)$$

where Cp is the concentrations of particle phase PAEs (ng/m^3), and V_d is the deposition velocity (cm/s), which can be used to estimate the deposition fluxes of air pollutants. In this work, we used the dry deposition velocity of 0.2 cm/s, which was estimated for pollutants concentrated in fine particles over the South China Sea.[8]

Text S5. Pearson correlation analysis

A correlation analysis of PAEs in air and seawater was conducted. Pearson correlation coefficient values of DiBP and DnBP ($p < 0.01$, $r \geq 0.951$), and DMP and DEP ($p < 0.01$, $r \geq 0.647$) (Table S14) indicated significant correlations between them, and can be attributed to similar sources and transport pathways. DEHP did not show any correlation with other PAEs. In seawater, DEP was highly correlated with DMP ($p < 0.01$, $r \geq 0.659$), BBP ($p < 0.01$, $r \geq 0.578$) and DEHP ($p < 0.01$, $r \geq 0.578$) (Table S15), and DEHP had a correlation with most other PAEs, except from DCHP. We suppose that PAEs have uniform sources for the seawater, while PAEs in air might be affected by their original sources, monsoon, and atmospheric degradation and precipitation.

Figure S1. Air sampling stations in the South China Sea, with the blue dots marked at the starting position of each air sample

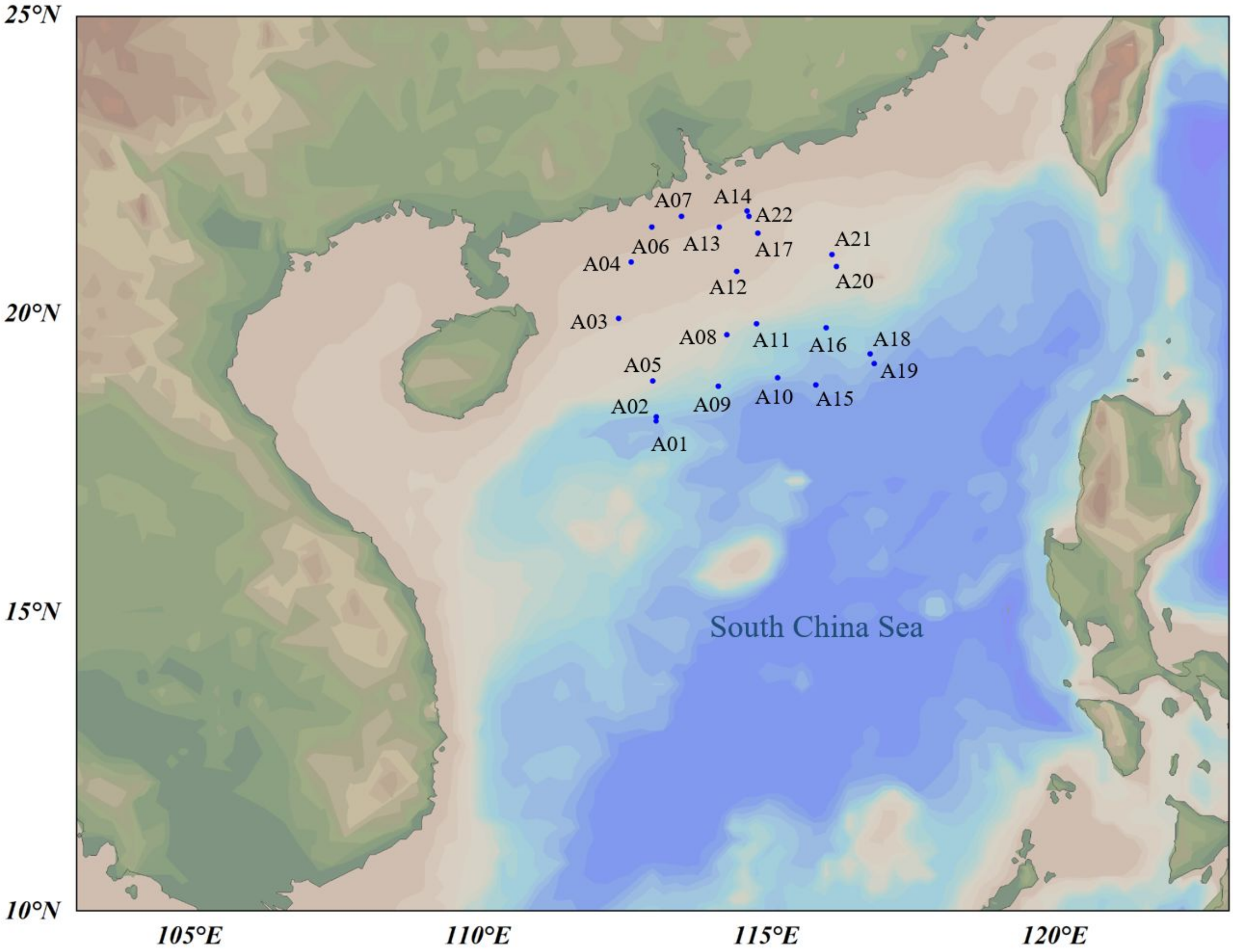

Figure S2. (A) Seawater sampling stations in the South China Sea, with the black dots marked at the starting position of each water sample. The color map shows the salinity profile in surface seawater; (B) general water mass circulation in the research area in summer

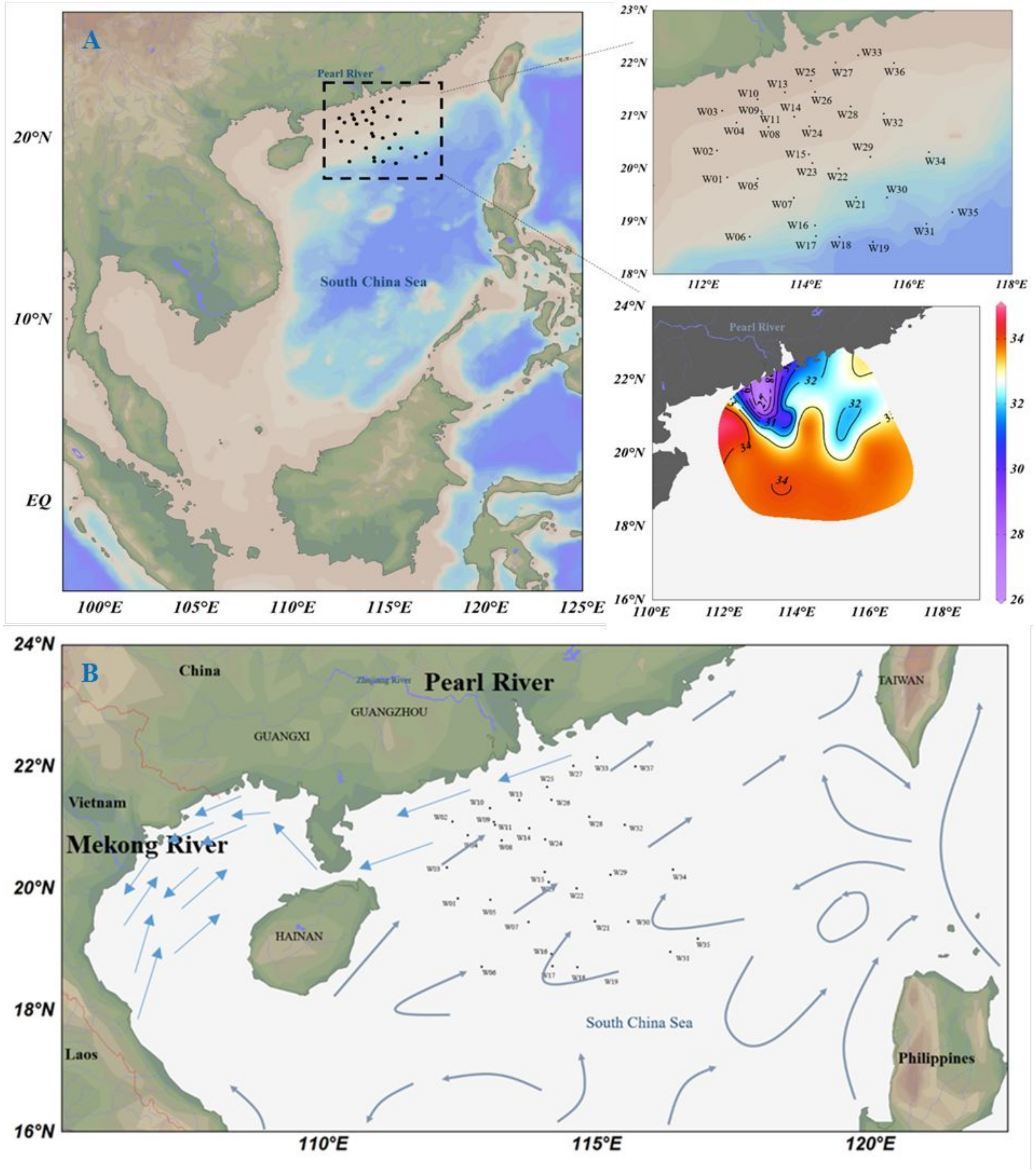

Figure S3A. High-volume air sampler is equipped with quartz fiber filter for atmospheric particles and a PUF/XAD-2 column for gaseous phase. The air sampler is placed at the upper deck and operated with headwind. The Filter and PUF-XAD-2 column are changed at sampling site. The PUF/XAD-2 column can be sealed with glass caps immediately after disconnected from the sampler to eliminate exposure time to the indoor air. The Quartz fiber filter is wrapped in a clean aluminum. PUF/XAD2 columns and filters are stored at -20°C;

S3B: The set-up of high-volume water sampler equipped with glass fiber filter and a XAD-2 column. The high-volume water sampler is connected to the ship-intake system with metal tubing. A glass fiber filter is closed in the filter holder to collect suspended particular matters and a XAD-2 column to retain organic chemicals in the dissolved phase. The seawater flows over the entire system at a flow rate of 0.2-1 L/min. The XAD-2 column is sealed with clips immediately after the sampling, and stored at 5°C. Glass fiber filter is wrapped in a clean aluminum foil and stored at -20°C

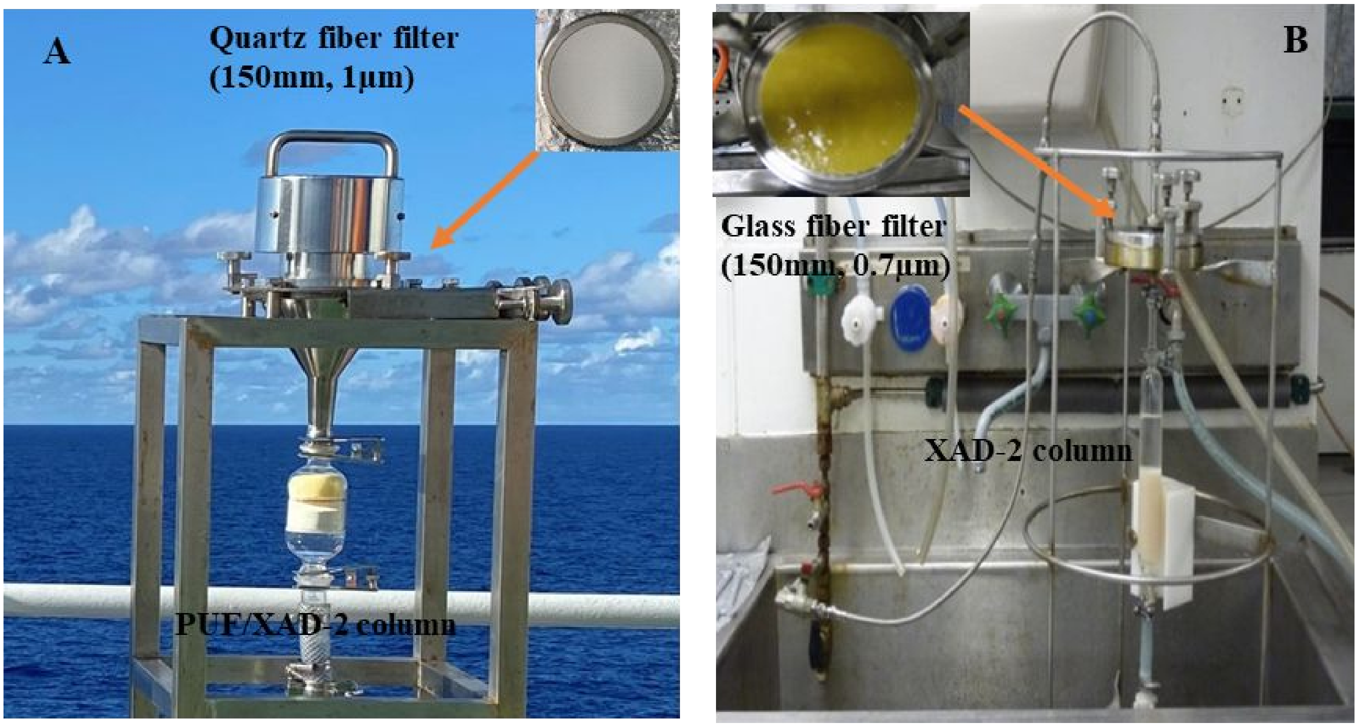

Figure S4**.** Air mass back trajectories of air samples. Colors indicate the origin of the air mass parcels

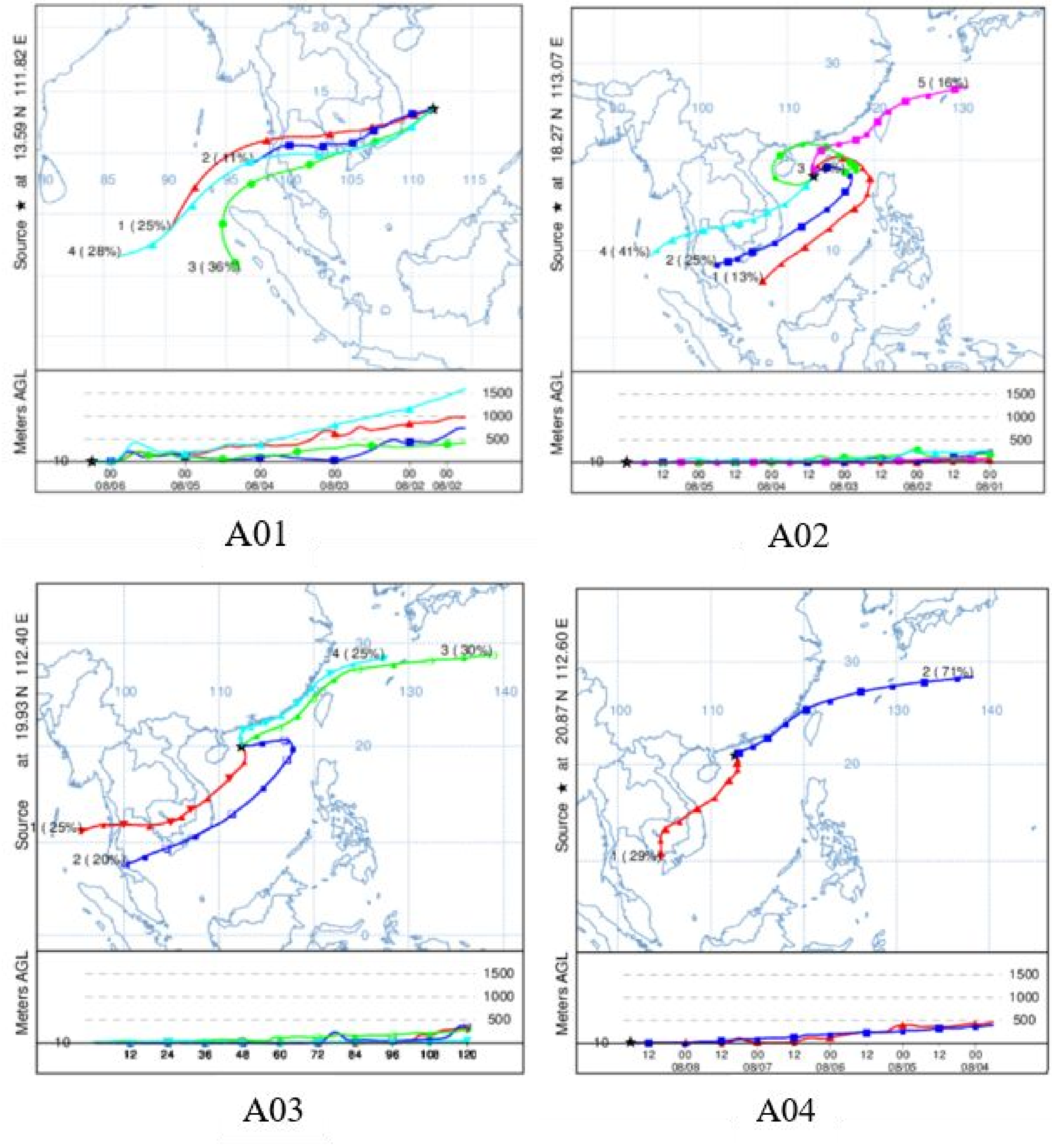

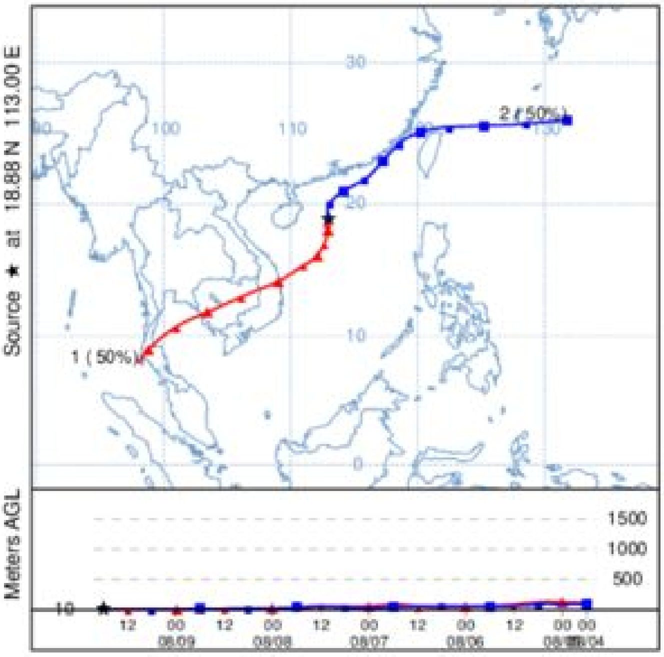

A05

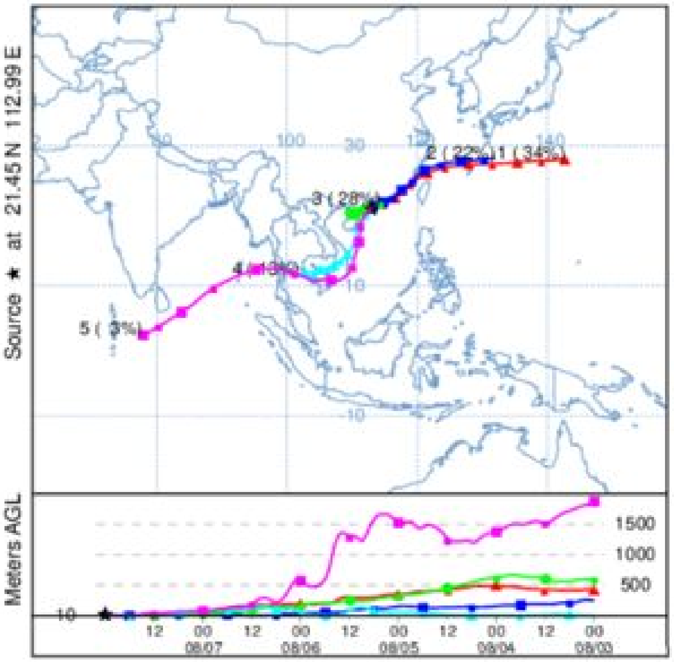

A06

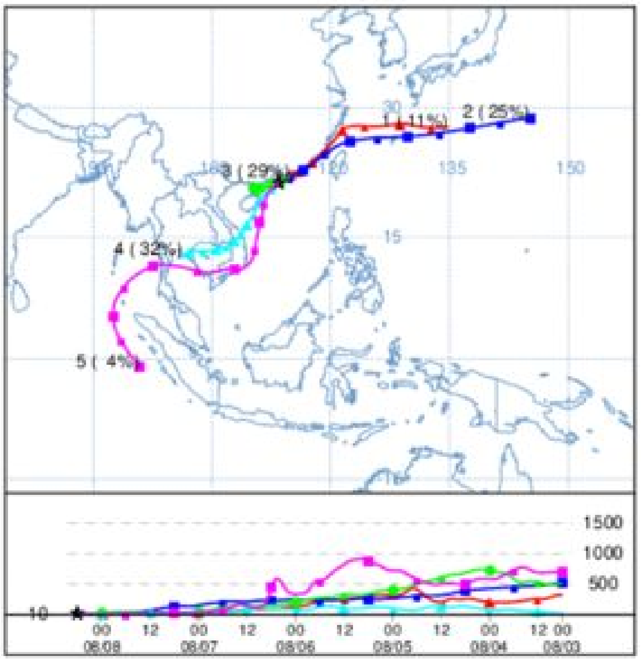

A07

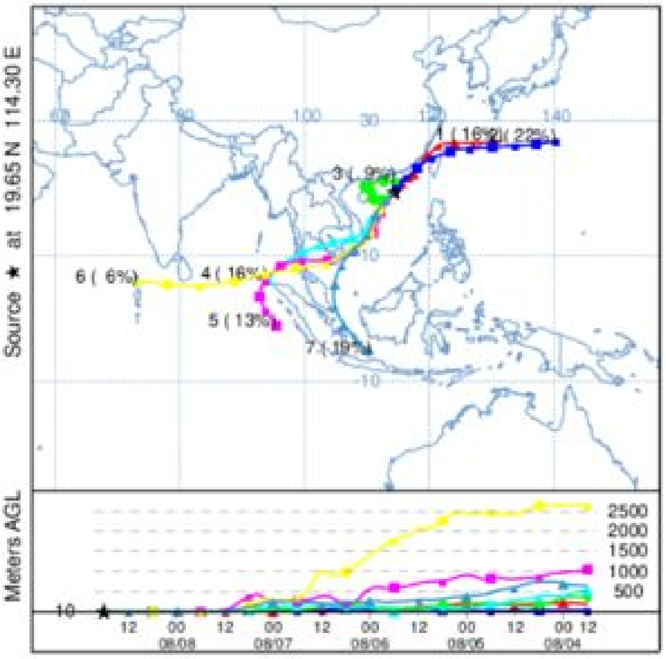

A08

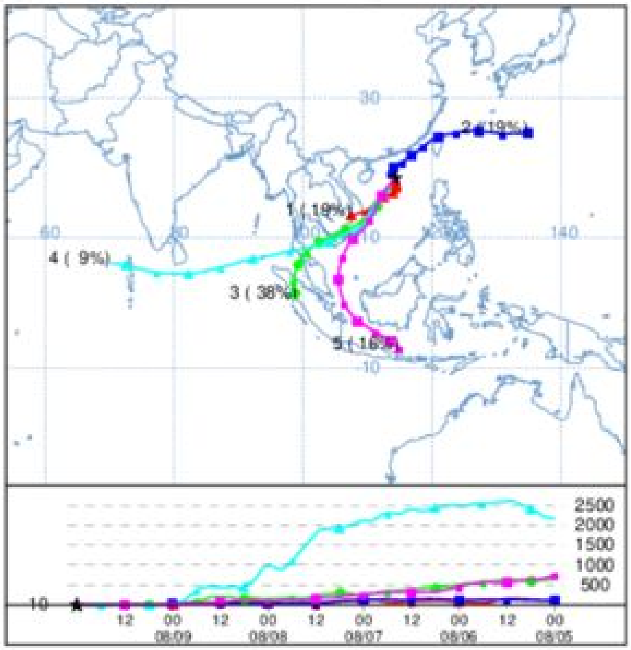

A09

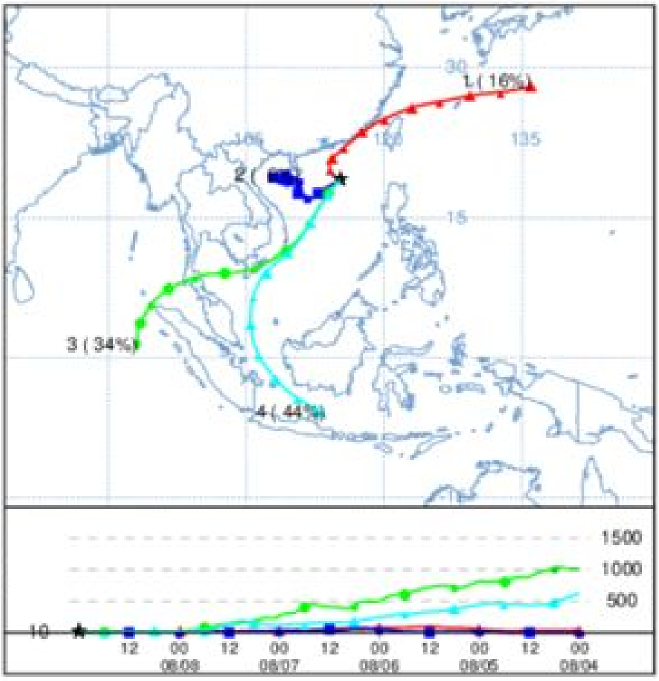

A10

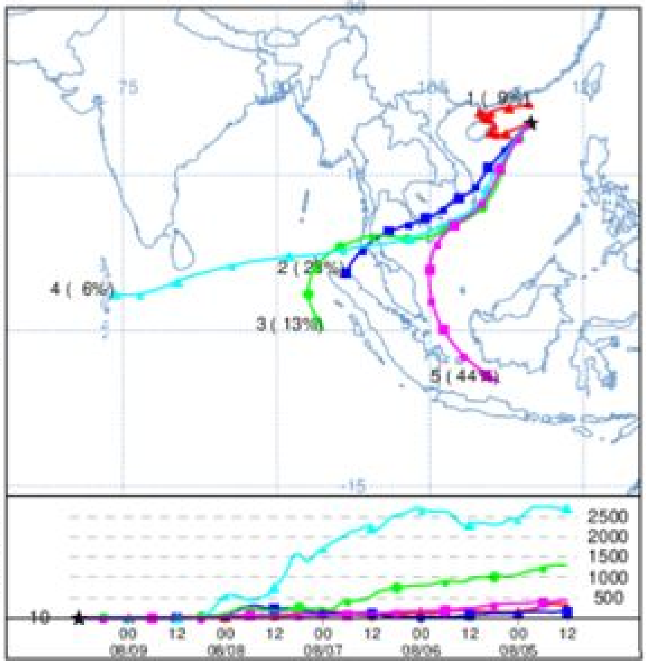

A11

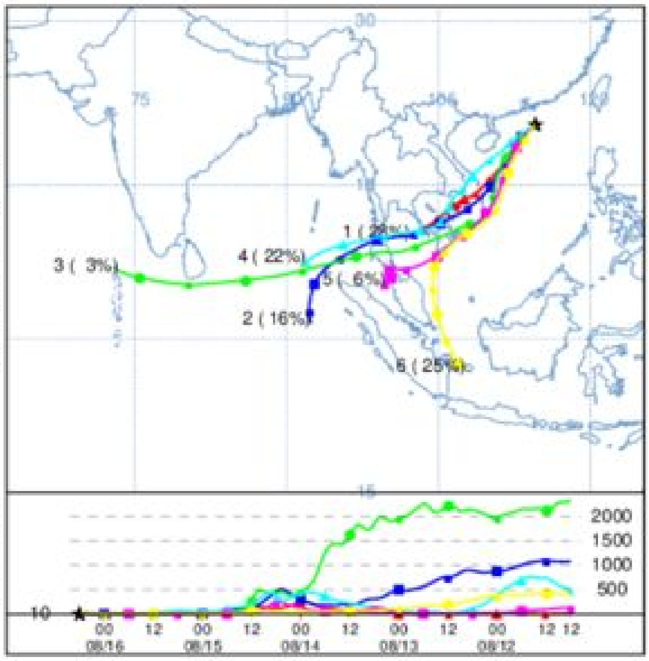

A12

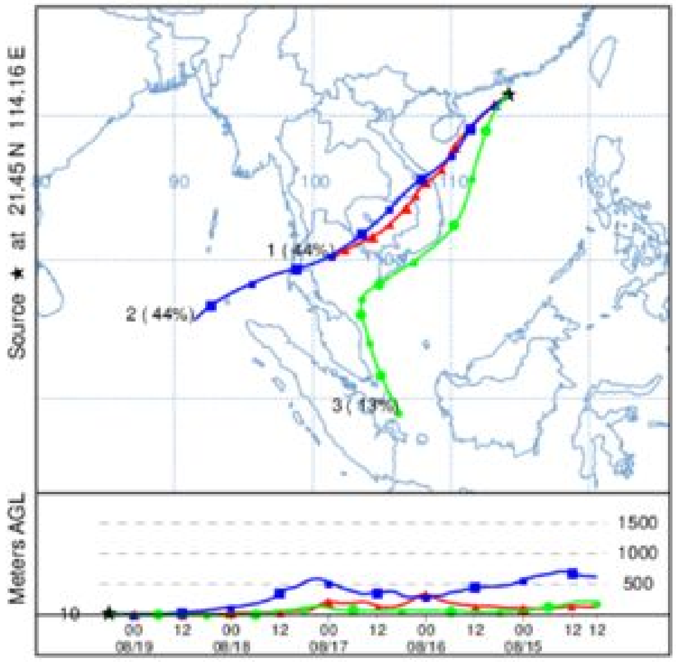

A13

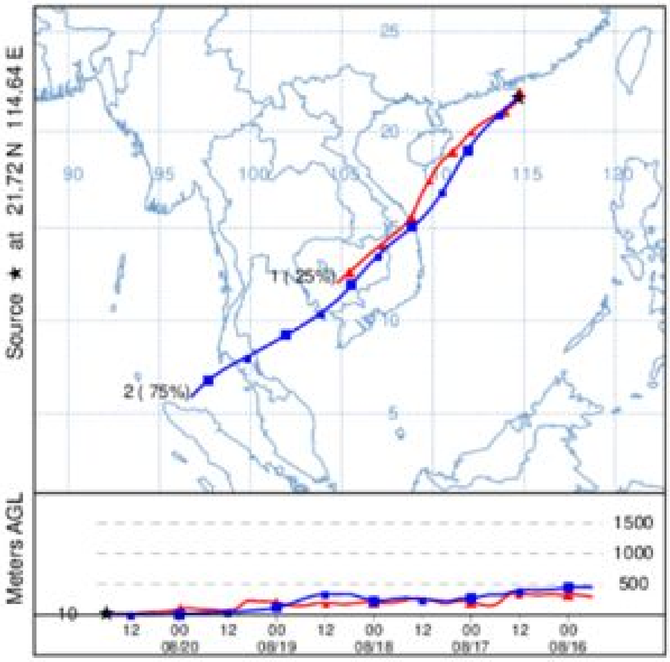

A14

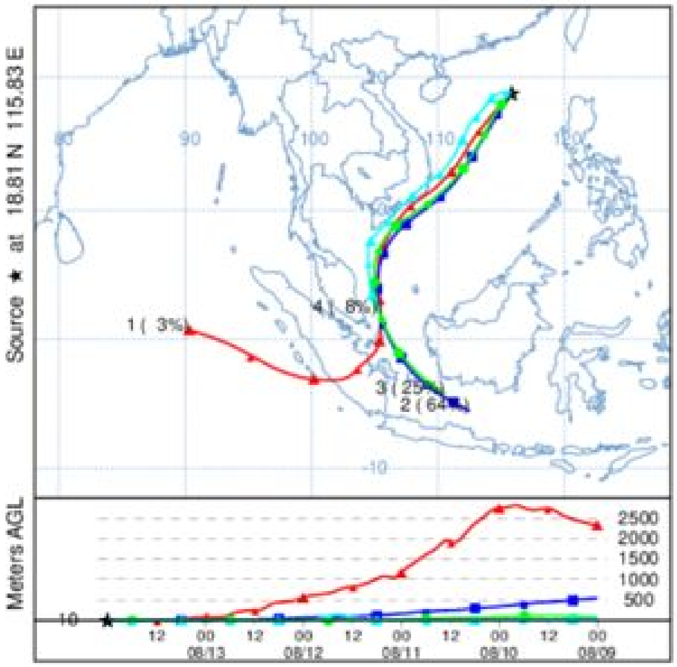

A15

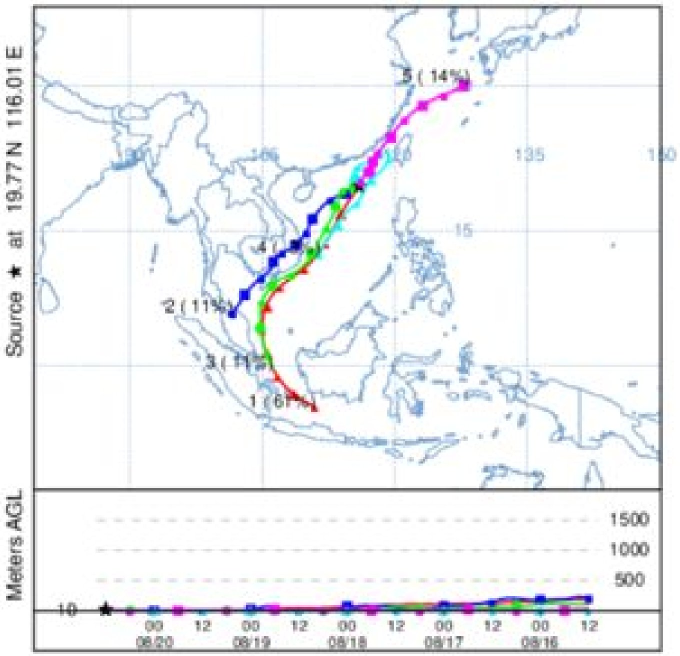

A16

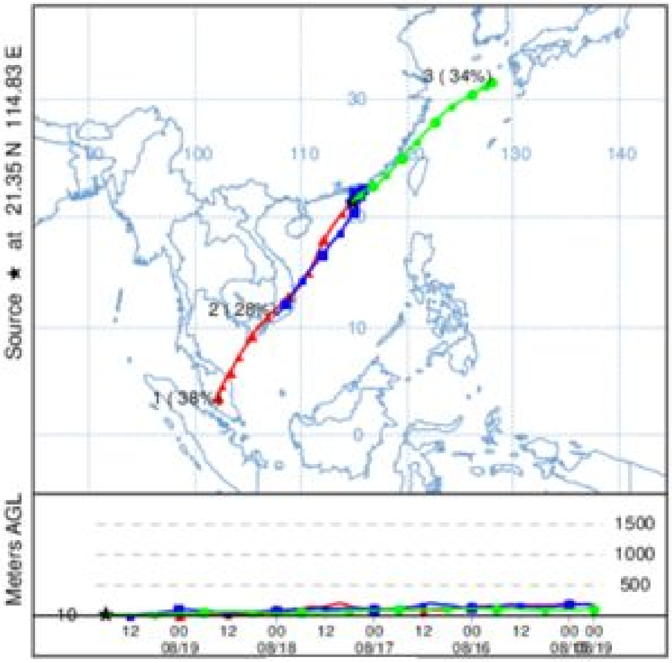

A17

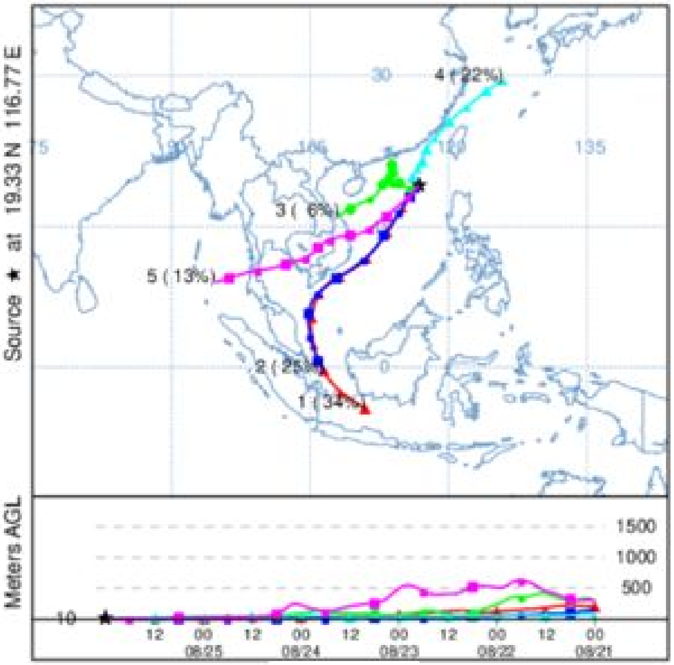

A18

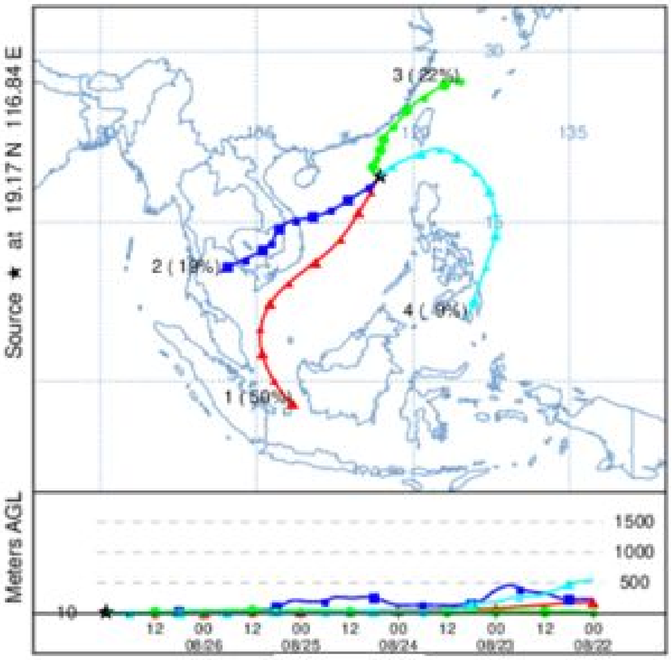

A19

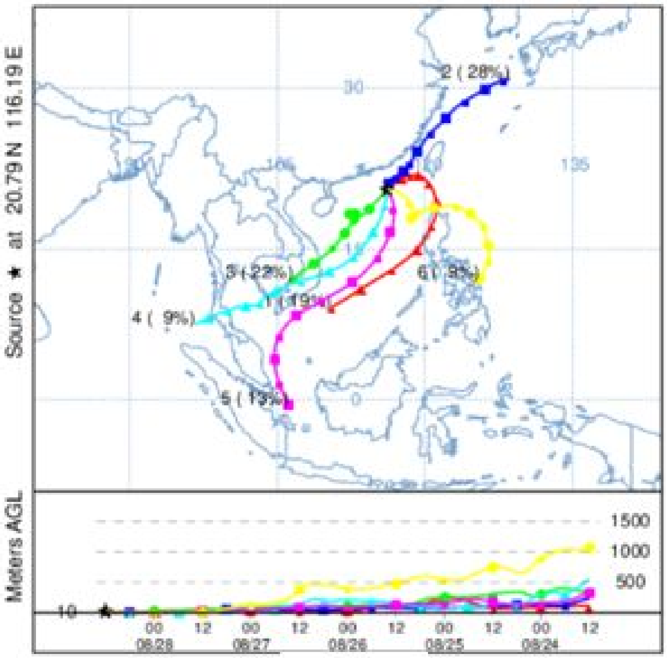

A20

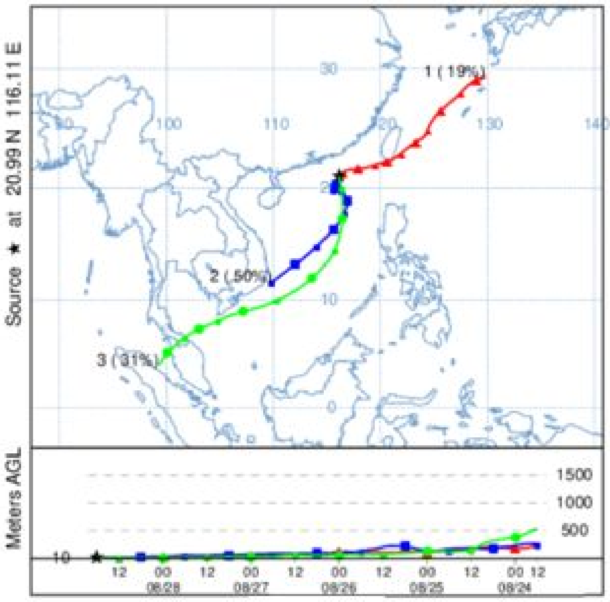
Source ★ at 20.99 N 116.11 E
1 (19%)
2 (50%)
3 (31%)
Meters AGL
1500
1000
500
10
08/28
08/27
08/26
08/25
08/24

A21

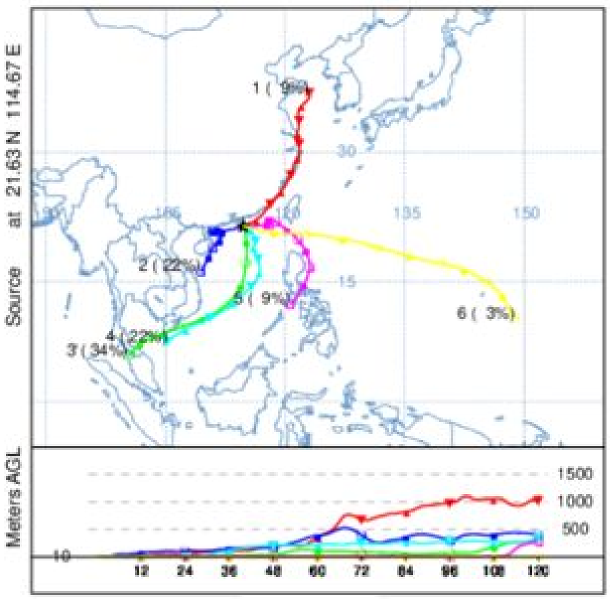
Source at 21.63 N 114.67 E
2 (22%)
4 (22%)
3 (34%)
5 (9%)
6 (3%)
Meters AGL
1500
1000
500
10
12
24
36
48
60
72
84
96
108
120

A22

Figure S5. The schematic diagrams of the flow field of the South China Sea in 7th (A) 13th (B), 19th (C) and 25th (D) of August 2019

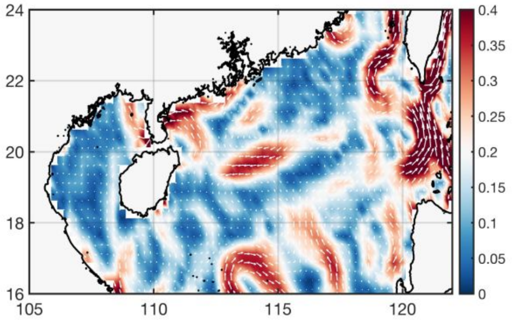

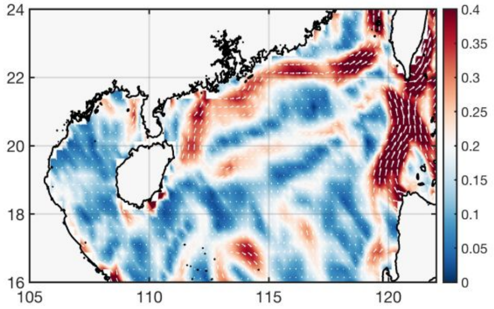

B

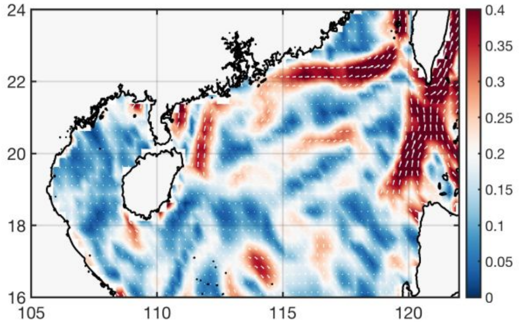

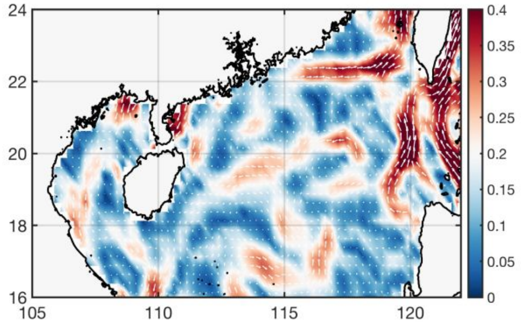

D

Table S 1. Detail information for air sampling

Air sample	Latitude (North, degree)	Longitude (East, degree)	Sampling Date	Time	Volume (m^3)	Air temperature (℃)	Humidity (%)	Wind speed (m/s)	Wind direction (degree)
**A01**	13.589	111.820	5-Aug-2019	0:37	382	29.1	73.8	11.3	244.5
**A02**	18.278	113.070	6-Aug-2019	7:30	373	29	82.4	9.9	336.8
**A03**	19.927	112.414	7-Aug-2019	11:08	293	28.8	83.7	7.2	50.2
**A04**	20.867	112.632	8-Aug-2019	10:37	269	29.2	80.8	4.8	232.2
**A05**	18.875	112.997	9-Aug-2019	11:28	326	29.5	82.7	6.3	205
**A06**	21.453	112.986	11-Aug-2019	11:03	341	29.6	83.4	8.2	219.5
**A07**	21.631	113.505	12-Aug-2019	11:03	348	29.6	84.5	8.2	221.6
**A08**	19.651	114.290	13-Aug-2019	10:12	344	29.6	84.2	8.4	219.1
**A09**	18.787	114.142	14-Aug-2019	10:54	280	29.7	75.3	8.9	229.6
**A10**	18.926	115.167	15-Aug-2019	10:54	316	29.2	82.8	11.1	201.3
**A11**	19.839	114.797	16-Aug-2019	10:54	278	29.6	83	7.5	198.8
**A12**	20.708	114.456	17-Aug-2019	11:05	645	29.4	83.8	7.6	214.4
**A13**	21.452	114.160	19-Aug-2019	10:39	290	29.4	74.8	6	223
**A14**	21.720	114.644	20-Aug-2019	11:00	535	28.6	74.4	8.3	94.3
**A15**	18.813	115.830	22-Aug-2019	10:39	339	26.6	87	9.7	314.9
**A16**	19.766	116.008	23-Aug-2019	11:46	356	28.9	80.3	3.4	26
**A17**	21.351	114.831	24-Aug-2019	11:46	504	30.3	79.9	12.5	242.3
**A18**	19.332	116.770	26-Aug-2019	11:00	310	29.2	81.7	3.9	141.6
**A19**	19.166	116.840	27-Aug-2019	11:00	531	28.7	81.7	7.2	35.3
**A20**	20.791	116.190	29-Aug-2019	11:00	345	28.5	81.3	7.8	124.7
**A21**	20.993	116.111	30-Aug-2019	11:06	313	28.5	78.1	4.6	108.8
**A22**	21.630	114.665	31-Aug-2019	11:15	300	28.7	79.4	7.9	81.5

Table S 2. Detail information for seawater sampling

Seawater Sample	Latitude (North, degree)	Longitude (East, degree)	Sampling Date	Volume (L)	Ta (℃)	Tw (℃)	Conductivity (S/m)	Depth (m)
**W01**	19.83	112.45	7-Aug-2019	100	28.8	29.9	5.64	113.2
**W02**	20.34	112.25	7-Aug-2019	135	28.5	29.6	5.65	83.4
**W03**	21.09	112.35	8-Aug-2019	171	29	29.4	5.64	48.8
**W04**	20.87	112.63	8-Aug-2019	178	29.4	29.6	5.67	60
**W05**	19.81	113.04	9-Aug-2019	193	29.4	30	5.67	154
**W06**	18.71	112.89	9-Aug-2019	177	29.4	30	5.67	900
**W07**	19.45	113.75	10-Aug-2019	244	29.8	29.8	5.67	472.5
**W08**	20.78	113.25	10-Aug-2019	196	29.3	30	5.4	81.1
**W09**	21.09	113.12	11-Aug-2019	36	29.8	30.3	4.74	60
**W10**	21.31	113.04	11-Aug-2019	169	29.8	29.8	4.57	42
**W11**	21.04	113.13	12-Aug-2019	84	30.3	30.4	4.98	44.3
**W12**	21.04	113.13	12-Aug-2019	119	30.3	30.4	4.98	44.3
**W13**	21.44	113.57	12-Aug-2019	68	29.7	29.6	5.18	42.2
**W14**	20.98	113.75	12-Aug-2019	105	29.7	30.4	4.83	85.6
**W15**	20.27	114.04	13-Aug-2019	310	29.8	29.6	5.58	121.4
**W16**	18.92	114.16	13-Aug-2019	199	29.5	29.8	5.66	1348.3
**W17**	18.72	114.18	14-Aug-2019	198	29.5	29.8	5.66	1859.1
**W18**	18.70	114.64	14-Aug-2019	126	29.2	29.7	5.65	3510
**W19**	18.61	115.29	15-Aug-2019	247	29.2	29.4	5.62	3694.5
**W21**	19.45	114.96	16-Aug-2019	195	29.9	29.7	5.62	1469.2
**W22**	20.00	114.62	16-Aug-2019	195	27.7	29.7	5.58	1469.2
**W23**	20.10	114.11	17-Aug-2019	165	29.6	29.8	5.61	187.5
**W24**	20.80	114.05	18-Aug-2019	165	29.6	30	5.68	89
**W25**	21.66	114.08	18-Aug-2019	217	27.7	29.6	5.45	51
**W26**	21.45	114.16	19-Aug-2019	210	29.2	29.9	5.42	55
**W27**	22.01	114.56	20-Aug-2019	143	28.4	29.2	5.3	40
**W28**	21.17	114.85	20-Aug-2019	158	28.4	29.7	5.46	95
**W29**	20.22	115.24	21-Aug-2019	185	26.7	29.4	5.35	468
**W30**	19.45	115.56	22-Aug-2019	258	28.7	29.3	5.56	2490
**W31**	18.95	116.33	23-Aug-2019	240	28.8	29.4	5.61	2732
**W32**	21.04	115.5	24-Aug-2019	220	29.4	29.8	5.34	125
**W33**	22.14	115	25-Aug-2019	250	27.5	29.9	5.44	51.8
**W34**	20.31	116.38	26-Aug-2019	138	29.2	29.4	5.58	961
**W35**	19.17	116.84	26-Aug-2019	180	29.2	29.5	5.6	3339
**W36**	19.17	116.84	27-Aug-2019	35	29.5	29.6	5.6	3340
**W37**	22.00	115.7	31-Aug-2019	100	29.2	29.9	5.54	80

Table S 3. Physiochemical properties of seven phthalate esters[9]

Chemical Name	Abbreviation	CAS	MW	Chemical formula	V_p (Pa)	S (mg/L)	H (pa.m^3/mol)	logK_{ow}	logK_{oa}	logKaw	Half-life time in air (d)	Chemical structure
**Dimethyl phthalate**	DMP	131-11-3	194.19	$C_{10}H_{10}O_4$	0.263	5220	$9.78x10^{-3}$	1.61	7.01	-5.4	14.4	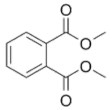
**Diethyl phthalate**	DEP	84-66-2	222.24	$C_{12}H_{14}O_4$	$6.48x10^{-2}$	591	$2.44x10^{-2}$	2.54	7.55	-5.01	2.39	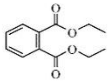
**Diisobutyl phthalate**	DiBP	84-69-5	278.35	$C_{16}H_{22}O_4$	$4.73x10^{-3}$	9.9	0.13	4.27	8.54	-4.27	0.58	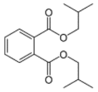
**Dibutyl phthalate**	DnBP	84-74-2	278.34	$C_{16}H_{22}O_4$	$4.73x10^{-3}$	9.9	0.13	4.27	8.54	-4.27	0.89	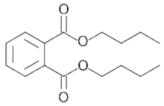
**Benzylbutyl phthalate**	BBP	85-68-7	312.37	$C_{19}H_{20}O_4$	$2.49x10^{-3}$	3.8	0.205	4.70	8.78	-4.08	0.75	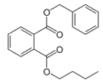
**Dicyclohexyl phthalate**	DCHP	84-61-7	330.42	$C_{20}H_{26}O_4$	$6.1x10^{-4}$	$4.1x10^{-2}$	4.92	6.2	11.59	-5.39	0.22	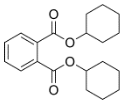
**Bis(2-ethylhexyl) phthalate**	DEHP	117-81-7	390.56	$C_{24}H_{38}O_4$	$2.52x10^{-5}$	$2.49x10^{-3}$	3.95	7.73	10.53	-2.80	0.38	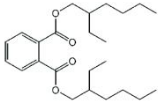

V_p: vapor pressure at 25°C; S: water solubility at 25°C; H: Henry's law constants at 25°C; logKow: log Octanol-Air coefficient at 25°C; logK_{oa}: log Octanol-Air coefficient at 25°C; logKaw: log air-water transfer coefficient; Half-life time in air (d) (exclude DiBP and DCHP)[10]; Half-live time in air for DiBP and DCHP: (Calculated by EPIWEB 4.1). Physiochemical properties of DCHP was Calculated with EPIWEB 4.1.

Table S 4. Information about the chemicals and material applied in this work

Chemical and material	Description	Supplier
Dichloromethane	Pico grade	Promochem, Wesel, Germany
n-Hexane	Pico grade	Promochem, Wesel, Germany
Methanol	Pico grade	Promochem, Wesel, Germany
Acetone	Pico grade	Promochem, Wesel, Germany
Dimethyl phthalate-3,4,5,6-d4	Ring-d4, 98%, 100µg/mL	LGC, Wesel, Germany
Diethyl phthalate-3,4,5,6-d4	Ring-d4, 98%, 100µg/mL	LGC, Wesel, Germany
Dibutyl phthalate-3,4,5,6-d4	Ring-d4, 98%, 100µg/mL	LGC, Wesel, Germany
Bis(2-ethylhexyl)phthalate-3,4,5,6-d4	Ring-d4, 98%, 100µg/mL	LGC, Wesel, Germany
2, 2',3,3',4,5,5',6,6'-Nonachlorobiphenyl (13C12)	99%, 2µg/mL	Cambridge Isotope Laboratories, UK
Phthalate esters Analytes Mix 3 Content: DMP, DEP, DiBP, DnBP, BBP, DCHP, DEHP and other 10 PAEs	1000µg/mL	LGC, Wesel, Germany
Silica gel	0.063-0.2mm	Macherey Nagel, Düren, Germany
Anhydrous sodium sulphate	99%	Merck, Darmstadt, Germany
Amberlite XAD-2	20-60 mesh	Merck, Darmstadt, Germany

Table S 5. Agilent 7890 GC-MS/MS conditions

Parameter	Value
Analytical column	2 set Agilent HP5-ms, 250 μm × 15 m, 0.25 μm
Sample injection mode	1 μL Pulsed splitless using Multimode Inlet (MMI)
Injection port liner	Agilent 200 μL dimpled, single-taper liner
Injection temperature program	20 °C (0.2 minutes), 300 °C/min to 300 ℃
Injection pulsed pressure	25 psi (1.9 minutes)
Purge flow to spit vent	50 mL/min (2.0 minutes)
Carrier gas	Helium
Carrier gas flow	1.2 mL/min
Oven temperature program	50 °C for 2 min, 20 °C/min to 80 °C, 5 °C/min to 250 °C, 15 °C/min to 300 °C (14 min)
Post run	310 °C for 3 min
Backflush	3.0 ml/min
Mass spectrometer	Agilent 7000C with electron impact ionization source operated in multiple reaction monitoring mode (MRM)
Ionization mode	Positive
Source temperature	280 °C
Quadrupole 1 and 2 temperature	150 °C
Transfer line temperature	280 °C

Table S 6. Detail information for the determination of PAEs using GC-MS/MS

PAE	Quantifier ions (m/z)	Qualifier ions (m/z)	Collision energy (eV)	Retention time (min)	Instrumental detection limit(pg)
**DMP**	163>77	163>135	15	16.963	0.02
**DEP**	149>93	149>121	15	20.258	0.03
**DiBP**	149>93	149>121	15	26.105	0.04
**DnBP**	149>93	149>121	15	27.956	0.04
**BBP**	149>93	149>121	15	34.854	0.1
**DCHP**	149>93	149>121	15	37.532	0.1
**DEHP**	149>93	149>121	15	37.955	0.2
**d4-DMP**	167>81	167>139	15	16.933	-
**d4-DEP**	153>97	153>125	15	20.232	-
**d4-DnBP**	153>97	153>125	15	27.937	-
**d4-DEHP**	153>97	153>125	15	37.943	-

Table S 7. The field blanks and the method detection limits of PAEs in PUF/XAD-2, XAD-2 columns, QFF and GFF filters

**Sample material**	**PUF/XAD-2 column (gas)**		**QFF (particle)**		**XAD-2 column (dissolved phase)**		**GFF (particular matter)**	
**PAE**	**Blank (mean±SD) (ng/m^3)**	**MDL (ng/m^3)**	**Blank (mean±SD) (ng/m^3)**	**MDL (ng/m^3)**	**Blank (mean±SD) (ng/L)**	**MDL (ng/L)**	**Blank (mean±SD) (ng/L)**	**MDL (ng/L)**
**DMP**	0.017±0.006	0.036	0.002±0.001	0.004	0.002±0.001	0.004	0.005±0.002	0.011
**DEP**	0.014±0.002	0.022	0.008±0.004	0.021	0.008±0.004	0.021	0.021±0.008	0.045
**DiBP**	0.020±0.007	0.042	0.005±0.001	0.007	0.005±0.001	0.007	0.045±0.011	0.077
**DnBP**	0.037±0.011	0.068	0.006±0.002	0.011	0.006±0.002	0.011	0.015±0.001	0.017
**BBP**	0.060±0.010	0.090	0.001±0.000	0.001	0.001±0.000	0.001	0.001±0.001	0.002
**DCHP**	0.001±0.000	0.002	0.001±0.000	0.001	0.001±0.000	0.001	0.001±0.000	0.001
**DEHP**	0.14±0.002	0.15	0.074±0.031	0.166	0.074±0.031	0.17	0.17±0.099	0.47

Table S 8. Concentrations of PAEs in the atmosphere over the South China Sea. The MDLs of PAEs calculated with individual sampling volumes are given in brackets for the calculated concentrations less than MDLs.

Air sample	DMP	DEP	DiBP	DnBP	BBP (MDL)	DCHP (MDL)	DEHP	$\sum_7$PAEs
**Gaseous phase (Cg, ng/m^3)**								
**A01**	0.45	0.10	1.37	0.94	0.02 (0.09)	0.00 (0.002)	2.80	5.68
**A02**	0.26	0.25	0.74	0.34	0.06 (0.09)	0.00 (0.002)	1.42	3.07
**A03**	1.60	0.14	2.93	2.13	0.09 (0.11)	0.002	0.47	7.36
**A04**	0.89	0.17	3.46	2.48	0.03 (0.12)	0.024	0.86	7.91
**A05**	0.45	0.26	1.46	1.13	0.03 (0.10)	0.005	0.68	4.00
**A06**	0.27	0.13	0.58	0.57	0.07 (0.10)	0.001 (0.002)	0.67	2.28
**A07**	0.32	0.17	1.93	1.63	0.03 (0.09)	0.003	0.70	4.78
**A08**	0.15	0.06	0.29	0.35	0.05 (0.10)	0.001 (0.002)	0.46	1.37
**A09**	0.35	0.13	1.15	0.82	0.02 (0.12)	0.013	0.63	3.12
**A10**	0.19	0.06	0.15	0.09	0.03 (0.10)	0.001 (0.002)	0.25	0.76
**A11**	0.29	0.26	1.76	1.19	0.01 (0.12)	0.001 (0.003)	0.50	4.00
**A12**	0.28	0.14	4.02	2.90	0.08	0.006	0.36	7.79
**A13**	0.68	0.15	2.31	1.81	0.08 (0.11)	0.002 (0.003)	0.85	5.88
**A14**	0.65	0.15	3.58	3.00	0.03 (0.06)	0.004	0.79	8.21
**A15**	0.36	0.05	0.46	0.38	0.02 (0.10)	0.002	0.31	1.58
**A16**	1.08	0.12	0.05	0.15	0.04 (0.09)	0.001 (0.002)	0.22	1.68
**A17**	1.53	0.39	1.50	1.96	0.02 (0.06)	0.002	1.08	6.48
**A18**	0.12	0.06	1.10	0.78	0.07 (0.10)	0.001 (0.002)	0.19	2.32
**A19**	0.14	0.06	1.30	1.12	0.04 (0.06)	0.003	0.34	3.00
**A20**	0.24	0.08	0.16	0.12	0.01 (0.10)	0.00 (0.002)	0.22	0.83
**A21**	0.47	0.19	1.26	1.06	0.05 (0.10)	0.002	0.30	3.33
**A22**	0.25	0.20	4.63	4.26	0.07 (0.10)	0.012	0.55	9.98
**Mean**	0.50	0.15	1.64	1.33	0.04 (0.09)	0.00 (0.002)	0.67	4.34
**S.D.**	0.42	0.08	1.33	1.10	0.03	0.01	0.57	2.70
**Median**	0.34	0.14	1.33	1.09	0.03 (0.09)	0.00 (0.002)	0.52	3.67
**Max**	1.60	0.39	4.63	4.26	0.09 (0.09)	0.02	2.80	9.98
**Min**	0.12	0.05	0.05	0.09	0.01 (0.09)	0.00 (0.002)	0.19	0.76
**Particle phase (Cp, ng/m^3)**								
**A01**	0.01	0.02	0.01	0.02	0.0002 (0.001)	0.000 (0.001)	0.54	0.60
**A02**	0.01	0.01	0.10	0.06	0.001	0.000 (0.001)	1.15	1.32
**A03**	0.09	0.21	8.08	5.31	0.001	0.0013	1.04	14.72
**A04**	0.03	0.07	6.35	3.60	0.002	0.0102	0.49	10.56
**A05**	0.02	0.08	5.08	2.74	0.000 (0.001)	0.0011	0.60	8.52
**A06**	0.02	0.04	2.94	3.41	0.003	0.000 (0.001)	0.39	6.81
**A07**	0.01	0.04	0.36	1.65	0.005	0.0079	0.40	2.47
**A08**	0.01	0.04	0.96	1.24	0.002	0.0025	0.31	2.56
**A09**	0.02	0.07	2.87	2.63	0.002	0.0139	0.39	6.00
**A10**	0.02	0.05	7.97	6.57	0.005	0.0015	0.73	15.34
**A11**	0.01	0.05	3.05	3.54	0.003	0.0043	0.53	7.20
**A12**	0.01	0.02	0.31	0.33	0.002	0.0012	0.23	0.90
**A13**	0.03	0.03	0.36	0.48	0.001	0.0024	0.44	1.34
**A14**	0.02	0.07	0.10	0.24	0.004	0.0051	0.50	0.93
**A15**	0.02	0.14	3.20	2.38	0.008	0.0062	0.47	6.22
**A16**	0.16	0.14	0.13	0.17	0.003	0.001 (0.001)	0.55	1.16
**A17**	0.01	0.06	0.15	0.23	0.003	0.0010	0.60	1.06
**A18**	0.03	0.13	0.30	0.31	0.001	0.0005	0.68	1.46
**A19**	0.01	0.02	0.01	0.03	0.000 (0.001)	0.0008	0.15	0.21
**A20**	0.03	0.07	3.67	2.49	0.002	0.001 (0.001)	0.32	6.58
**A21**	0.04	0.20	10.50	9.44	0.005	0.020	0.80	21.00
**A22**	0.01	0.03	0.04	0.06	0.000 (0.001)	0.001 (0.001)	0.34	0.48
**Mean**	0.03	0.07	2.57	2.13	0.002	0.004	0.53	5.34
**S.D.**	0.03	0.06	3.17	2.47	0.002	0.005	0.24	5.73
**Median**	0.02	0.06	0.66	1.44	0.002	0.001	0.50	2.51
**Max**	0.16	0.21	10.50	9.44	0.008	0.020	1.15	21.00
**Min**	0.00	0.01	0.01	0.02	0.000 (0.001)	0.000 (0.001)	0.15	0.21
**Sum of Cg+Cp (ng/m^3)**								
**A01**	0.46	0.12	1.38	0.96	0.02	0.00	3.34	6.28
**A02**	0.26	0.26	0.84	0.40	0.06	0.00	2.57	4.39
**A03**	1.69	0.35	11.01	7.44	0.09	0.00	1.51	22.08
**A04**	0.92	0.24	9.81	6.08	0.03	0.03	1.35	18.47
**A05**	0.47	0.34	6.54	3.87	0.03	0.00	1.28	12.52
**A06**	0.29	0.17	3.52	3.98	0.07	0.00	1.06	9.09
**A07**	0.33	0.21	2.29	3.28	0.03	0.01	1.10	7.25
**A08**	0.16	0.10	1.25	1.59	0.05	0.00	0.77	3.93

**A09**	0.37	0.20	4.02	3.45	0.02	0.02	1.02	9.12
**A10**	0.21	0.11	8.12	6.66	0.04	0.00	0.98	16.10
**A11**	0.30	0.31	4.81	4.73	0.01	0.00	1.03	11.20
**A12**	0.29	0.16	4.33	3.23	0.08	0.01	0.59	8.69
**A13**	0.71	0.18	2.67	2.29	0.08	0.00	1.29	7.22
**A14**	0.67	0.22	3.68	3.24	0.03	0.01	1.29	9.14
**A15**	0.38	0.19	3.66	2.76	0.03	0.01	0.78	7.80
**A16**	1.24	0.26	0.18	0.32	0.04	0.00	0.77	2.84
**A17**	1.54	0.45	1.65	2.19	0.02	0.00	1.68	7.54
**A18**	0.15	0.19	1.40	1.09	0.07	0.00	0.87	3.78
**A19**	0.15	0.08	1.31	1.15	0.04	0.00	0.49	3.21
**A20**	0.27	0.15	3.83	2.61	0.01	0.00	0.54	7.41
**A21**	0.51	0.39	11.76	10.50	0.05	0.02	1.10	24.33
**A22**	0.26	0.23	4.67	4.32	0.07	0.01	0.89	10.46
**Mean**	0.53	0.22	4.21	3.46	0.05	0.01	1.20	9.67
**S.D.**	0.44	0.10	3.31	2.48	0.02	0.01	0.66	5.86
**Median**	0.35	0.20	3.67	3.23	0.04	0.00	1.05	8.24
**Max**	1.69	0.45	11.76	10.50	0.09	0.03	3.34	24.33
**Min**	0.15	0.08	0.18	0.32	0.01	0.00	0.49	2.84

Table S 9. Particle-bound fractions (ø) of PAEs in air samples from the South China Sea. The particle-bound fractions are quite uncertain because BBP and DCHP were below MDLs in most particle samples.

Sample	DMP	DEP	DiBP	DnBP	BBP	DCHP	DEHP	PEs
**A01**	0.01	0.17	0.01	0.02	0.01	0.02	0.16	0.10
**A02**	0.02	0.05	0.11	0.16	0.01	0.49	0.45	0.30
**A03**	0.05	0.59	0.73	0.71	0.01	0.42	0.69	0.67
**A04**	0.03	0.29	0.65	0.59	0.06	0.30	0.36	0.57
**A05**	0.03	0.24	0.78	0.71	0.01	0.19	0.47	0.68
**A06**	0.07	0.23	0.84	0.86	0.04	0.29	0.37	0.75
**A07**	0.03	0.18	0.16	0.50	0.13	0.72	0.36	0.34
**A08**	0.06	0.40	0.77	0.78	0.03	0.65	0.40	0.65
**A09**	0.06	0.35	0.71	0.76	0.09	0.52	0.38	0.66
**A10**	0.08	0.42	0.98	0.99	0.15	0.72	0.75	0.95
**A11**	0.05	0.16	0.63	0.75	0.18	0.79	0.52	0.64
**A12**	0.03	0.10	0.07	0.10	0.02	0.17	0.40	0.10
**A13**	0.04	0.18	0.13	0.21	0.02	0.54	0.34	0.19
**A14**	0.03	0.32	0.03	0.07	0.13	0.54	0.39	0.10
**A15**	0.05	0.72	0.87	0.86	0.28	0.76	0.60	0.80
**A16**	0.13	0.54	0.70	0.52	0.06	0.36	0.72	0.41
**A17**	0.01	0.14	0.09	0.11	0.12	0.37	0.36	0.14
**A18**	0.2	0.68	0.22	0.29	0.02	0.42	0.78	0.39
**A19**	0.05	0.24	0.01	0.02	0.01	0.21	0.30	0.07
**A20**	0.1	0.46	0.96	0.95	0.13	0.86	0.59	0.89
**A21**	0.08	0.52	0.89	0.90	0.09	0.91	0.73	0.86
**A22**	0.05	0.13	0.01	0.01	0.01	0.06	0.38	0.05
**Mean**	0.06	0.32	0.47	0.49	0.07	0.47	0.48	0.47
**S.D.**	0.04	0.19	0.37	0.35	0.07	0.26	0.17	0.30

Table S 10. Washout ratio (W) calculated with particle bound fraction following eq. W = (1-ø)(RT/H)+øWp. BBP and DCHP were excluded because of low detection frequency.

Washout ratio (W)	DMP	DEP	DiBP	DnBP	DEHP
**A01**	250001	88055	18650	18661	3784
**A02**	249049	97693	18794	18851	9284
**A03**	241086	53620	19638	19610	13992
**A04**	246568	77786	19520	19444	7669
**A05**	245343	81678	19696	19603	9745
**A06**	237432	83066	19777	19805	7783
**A07**	245799	86685	18854	19323	7621
**A08**	238486	69124	19680	19701	8411
**A09**	239887	73298	19610	19677	8006
**A10**	235434	67580	19975	19983	15097
**A11**	242910	88639	19502	19658	10631
**A12**	246794	93344	18734	18777	8327
**A13**	244920	86717	18820	18923	7234
**A14**	247213	75085	18674	18738	8111
**A15**	241118	42584	19830	19812	12229
**A16**	222975	57545	19594	19352	14479
**A17**	251596	90011	18759	18782	7559
**A18**	207786	46031	18934	19031	15690
**A19**	241474	82131	18647	18670	6495
**A20**	229263	64022	19945	19937	12013
**A21**	235419	59487	19854	19862	14700
**A22**	242384	91108	18649	18656	8038
**Max**	251596	97693	19975	19983	15690
**Min**	207786	42584	18647	18656	3784
**Mean**	240134	75241	19279	19312	9859
**SD**	9895	15795	510	480	3261
**Median**	241929	79732	19511	19398	8369

Table S 11. Concentrations of PAEs in the seawater from the South China Sea. The MDLs of PAEs calculated with individual sampling volumes are given in brackets for the calculated concentrations less than MDLs.

Sample	DMP	DEP	DiBP	DnBP	BBP	DCHP	DEHP	PAEs
**PAE in dissolved phase (C_W, ng/L)**								
**W01**	0.20	0.46	0.25	1.08	0.014	0.001 (0.002)	1.53	3.54
**W02**	0.15	0.27	0.21	1.38	0.008	0.001 (0.001)	1.39	3.40
**W03**	0.41	0.30	0.14	0.73	0.006	0.001 (0.001)	0.77	2.36
**W04**	0.12	0.14	0.18	0.49	0.005	0.001 (0.001)	0.93	1.85
**W05**	0.20	0.25	0.09	0.30	0.004	0.000 (0.001)	1.32	2.16
**W06**	0.24	0.14	0.11	0.50	0.003	0.001 (0.001)	0.63	1.64
**W07**	0.13	0.26	0.09	0.28	0.003	0.000 (0.001)	0.98	1.74
**W08**	1.60	0.93	0.31	0.21	0.006	0.013	0.48	3.55
**W09**	1.14	0.84	0.25	1.02	0.107	0.001 (0.005)	3.52	6.88
**W10**	1.30	0.57	0.13	0.17	0.027	0.007	2.03	4.23
**W11**	1.16	0.77	0.20	0.43	0.012	0.001 (0.002)	2.68	5.25
**W12**	0.77	0.35	0.08	0.20	0.003	0.000 (0.001)	1.01	2.42
**W13**	1.30	0.98	0.08	0.20	0.005	0.000 (0.002)	2.87	5.43
**W14**	0.25	0.43	0.09	0.22	0.008	0.000 (0.002)	3.30	4.30
**W15**	0.14	0.22	0.05	0.13	0.003	0.000 (0.001)	0.70	1.23
**W16**	0.14	0.19	0.07	0.17	0.020	0.001 (0.001)	1.04	1.62
**W17**	0.17	0.14	0.08	0.24	0.003	0.000 (0.001)	0.65	1.29
**W18**	0.07	0.11	1.24	0.30	0.003	0.000 (0.001)	1.48	3.20
**W19**	0.07	0.72	0.12	0.20	0.001 (0.001)	0.000 (0.001)	0.66	1.76
**W21**	0.13	0.32	0.08	0.16	0.006	0.002	0.55	1.25
**W22**	0.13	0.53	0.09	0.24	0.018	0.003	0.46	1.48
**W23**	0.13	0.40	0.14	0.33	0.022	0.003	0.68	1.70
**W24**	0.40	0.40	0.20	0.49	0.009	0.001 (0.001)	0.68	2.18
**W25**	0.75	0.52	0.18	0.37	0.020	0.004	0.59	2.44
**W26**	0.47	0.46	0.04	0.03	0.001 (0.001)	0.000 (0.001)	0.39	1.40
**W27**	0.55	0.38	0.12	0.21	0.007	0.003	0.75	2.02
**W28**	0.18	0.14	0.08	0.10	0.005	0.002	0.62	1.12
**W29**	0.20	0.17	0.07	0.11	0.001 (0.001)	0.002	0.91	1.46
**W30**	0.10	0.15	0.06	0.13	0.004	0.012	0.66	1.12
**W31**	0.15	0.13	0.06	0.12	0.001 (0.001)	0.001 (0.001)	0.38	0.83
**W32**	0.35	0.15	0.10	0.16	0.005	0.001 (0.001)	0.49	1.26
**W33**	1.12	0.41	0.41	0.66	0.020	0.000 (0.001)	1.84	4.46
**W34**	0.37	0.19	0.19	0.27	0.002	0.000 (0.001)	0.89	1.91
**W35**	0.15	0.31	0.21	0.22	0.049	0.005	1.28	2.23
**W36**	0.47	0.56	0.49	0.58	0.007	0.007	2.32	4.44
**W37**	2.14	0.54	0.48	0.71	0.003	0.007	1.37	5.26
**Mean**	0.48	0.38	0.19	0.37	0.012	0.002	1.19	2.62
**SD**	0.51	0.24	0.21	0.30	0.019	0.003	0.83	1.52

**Median**	0.22	0.34	0.12	0.24	0.005	0.001 (0.001)	0.90	2.09
**Min**	0.07	0.11	0.04	0.03	0.001 (0.001)	0.000 (0.001)	0.38	0.83
**Max**	2.14	0.98	1.24	1.38	0.110	0.013	3.52	6.88
**PAE in particle phase (C_{spm}, ng/L)**								
**W01**	0.046	0.11	0.05 (0.13)	0.18	0.010	0.000 (0.002)	1.58	1.97 (1.06)
**W02**	0.030	0.09	0.07 (0.10)	0.20	0.011	0.000 (0.001)	0.92	1.32
**W03**	0.024	0.08	0.05 (0.08)	0.11	0.015	0.000 (0.001)	0.99	1.27
**W04**	0.030	0.08	0.03 (0.07)	0.07	0.008	0.000 (0.001)	2.28	2.50
**W05**	0.012	0.05	0.03 (0.07)	0.07	0.005	0.000 (0.001)	0.74	0.91
**W06**	0.038	0.08	0.05 (0.07)	0.16	0.009	0.000 (0.001)	0.64	0.98
**W07**	0.021	0.06	0.03 (0.05)	0.11	0.006	0.000 (0.001)	0.49	0.73
**W08**	0.019	0.05	0.02 (0.07)	0.23	0.026	0.002	0.57	0.92
**W09**	0.072	0.20 (0.21)	0.13 (0.36)	0.43	0.013	0.000 (0.005)	2.1 (2.2)	2.94
**W10**	0.018	0.04 (0.045)	0.11	0.11	0.015	0.001 (0.001)	0.39 (0.47)	0.69
**W11**	0.037	0.09 (0.09)	0.23	0.28	0.027	0.002 (0.002)	0.74 (0.95)	1.41
**W12**	0.017	0.04 (0.06)	0.02 (0.11)	0.03	0.018	0.003	0.43 (0.67)	0.57 (0.89)
**W13**	0.047	0.09 (0.11)	0.1 (0.19)	0.06	0.024	0.003	0.57(1.17)	0.91 (1.55)
**W14**	0.040	0.08	0.03 (0.12)	0.04	0.002 (0.003)	0.000 (0.002)	0.44 (0.76)	0.63 (1.0)
**W15**	0.007	0.02 (0.02)	0.01 (0.04)	0.01	0.001 (0.001)	0.000 (0.001)	0.12 (0.26)	0.17 (0.34)
**W16**	0.035	0.05	0.02 (0.07)	0.04	0.002 (0.002)	0.000 (0.001)	0.28 (0.40)	0.43 (0.53)
**W17**	0.038	0.06	0.01 (0.07)	0.08	0.001 (0.002)	0.000 (0.001)	0.28 (0.40)	0.47 (0.53)
**W18**	0.012 (0.015)	0.03 (0.06)	0.03 (0.10)	0.06	0.001 (0.003)	0.000 (0.001)	0.18 (0.63)	0.31 (0.84)
**W19**	0.003 (0.008)	0.01 (0.03)	0.02 (0.05)	0.01 (0.01)	0.000 (0.001)	0.000 (0.001)	0.12 (0.32)	0.16 (0.43)
**W21**	0.007 (0.010)	0.10	0.07 (0.07)	0.14	0.020	0.000 (0.001)	0.94	1.27
**W22**	0.008 (0.010)	0.09	0.09	0.13	0.017	0.000 (0.001)	0.61	0.95
**W23**	0.011 (0.010)	0.11	0.06 (0.08)	0.10	0.013	0.000 (0.001)	0.6	0.89
**W24**	0.011 (0.010)	0.10	0.06 (0.08)	0.10	0.013	0.000 (0.001)	0.61	0.89
**W25**	0.009 (0.009)	0.09	0.06 (0.06)	0.09	0.011	0.000 (0.001)	0.59	0.84
**W26**	0.004 (0.009)	0.06	0.04 (0.06)	0.08	0.009	0.000 (0.001)	0.33 (0.38)	0.52
**W27**	0.009 (0.013)	0.09	0.04 (0.09)	0.06	0.005	0.000 (0.001)	0.39 (0.56)	0.59 (0.74)
**W28**	0.012 (0.012)	0.08	0.04 (0.08)	0.06	0.006	0.000 (0.001)	0.33 (0.50)	0.53
**W29**	0.011 (0.010)	0.08	0.04 (0.07)	0.07	0.006	0.000 (0.001)	0.37 (0.43)	0.59
**W30**	0.005 (0.007)	0.05	0.03 (0.05)	0.04	0.004	0.000 (0.001)	0.43	0.55
**W31**	0.006 (0.008)	0.03 (0.03)	0.02 (0.05)	0.03	0.002	0.000 (0.001)	0.16 (0.33)	0.24 (0.44)
**W32**	0.004 (0.008)	0.04	0.02 (0.06)	0.03	0.003	0.000 (0.001)	0.19 (0.36)	0.28 (0.48)
**W33**	0.005 (0.007)	0.03 (0.03)	0.02 (0.05)	0.02	0.001 (0.001)	0.000 (0.001)	0.53	0.60
**W34**	0.013 (0.014)	0.06	0.03 (0.10)	0.04	0.003	0.000 (0.001)	0.26 (0.58)	0.41 (0.77)
**W35**	0.009 (0.009)	0.06	0.02 (0.07)	0.04	0.002 (0.002)	0.000 (0.001)	0.2 (0.44)	0.33 (0.59)
**W36**	0.190	0.28	0.15 (0.37)	0.15	0.004 (0.01)	0.000 (0.005)	1.01 (2.28)	1.79 (3.02)
**W37**	0.034	0.05 (0.05)	0.05 (0.13)	0.05	0.005	0.000 (0.002)	0.27 (0.80)	0.46 (1.06)
**Mean**	0.025	0.076	0.052 (0.077)	0.098	0.009	0.000 (0.001)	0.60	0.86
**SD**	0.032	0.049	0.045 (0.077)	0.085	0.007	0.001 (0.001)	0.49	0.62 (0.62)
**Median**	0.013	0.070	0.040 (0.077)	0.074	0.006	0.000 (0.001)	0.47 (0.47)	0.66

**Min**	0.003 (0.011)	0.013	0.009 (0.077)	0.011 (0.017)	0.000 (0.002)	0.000 (0.001)	0.12 (0.47)	0.16 (0.62)
**Max**	0.190	0.279	0.229	0.428	0.027	0.003	2.27	2.94
**Sum of C_W + C_{SPM} (ng/L)**								
**W01**	0.24	0.57	0.30	1.26	0.012	0.000 (0.003)	3.11	5.51
**W02**	0.18	0.36	0.27	1.58	0.009	0.000 (0.003)	2.31	4.73
**W03**	0.43	0.38	0.20	0.84	0.010	0.001 (0.002)	1.76	3.63
**W04**	0.15	0.22	0.21	0.56	0.006	0.000 (0.002)	3.20	4.35
**W05**	0.21	0.30	0.12	0.37	0.005	0.000 (0.002)	2.06	3.07
**W06**	0.28	0.23	0.16	0.67	0.006	0.001 (0.002)	1.28	2.62
**W07**	0.15	0.32	0.12	0.39	0.005	0.000 (0.001)	1.47	2.47
**W08**	1.62	0.98	0.33	0.44	0.016	0.008	1.05	4.47
**W09**	1.21	1.04	0.38	1.44	0.060	0.000 (0.009)	5.62	9.82
**W10**	1.32	0.61	0.24	0.28	0.021	0.004	2.42	4.92
**W11**	1.20	0.85	0.43	0.71	0.020	0.002 (0.004)	3.42	6.65
**W12**	0.79	0.40	0.09	0.23	0.011	0.002 (0.003)	1.45	2.99
**W13**	1.34	1.08	0.18	0.26	0.014	0.002 (0.005)	3.44	6.34
**W14**	0.29	0.51	0.12	0.26	0.005 (0.005)	0.000 (0.003)	3.74	4.93
**W15**	0.14	0.23	0.06	0.14	0.002 (0.002)	0.000 (0.001)	0.82	1.40
**W16**	0.17	0.24	0.09	0.21	0.011	0.000 (0.002)	1.32	2.06
**W17**	0.21	0.20	0.10	0.32	0.002 (0.003)	0.000 (0.002)	0.93	1.76
**W18**	0.08	0.14	1.27	0.36	0.002 (0.004)	0.000 (0.003)	1.66	3.51
**W19**	0.07	0.74	0.13	0.21	0.001 (0.002)	0.000 (0.001)	0.78	1.93
**W21**	0.14	0.42	0.15	0.30	0.013	0.001 (0.002)	1.49	2.52
**W22**	0.14	0.62	0.18	0.38	0.017	0.002 (0.002)	1.07	2.43
**W23**	0.14	0.51	0.20	0.43	0.017	0.001 (0.002)	1.27	2.59
**W24**	0.41	0.50	0.26	0.59	0.011	0.001 (0.002)	1.29	3.07
**W25**	0.76	0.61	0.24	0.46	0.016	0.002 (0.002)	1.18	3.28
**W26**	0.47	0.52	0.09	0.11	0.005	0.000 (0.002)	0.72	1.92
**W27**	0.56	0.47	0.16	0.26	0.006	0.002 (0.002)	1.14	2.61
**W28**	0.20	0.21	0.12	0.15	0.005	0.001 (0.002)	0.95	1.65
**W29**	0.21	0.25	0.11	0.19	0.004	0.001 (0.002)	1.28	2.05
**W30**	0.10	0.20	0.09	0.18	0.004	0.006	1.08	1.67
**W31**	0.16	0.16	0.08	0.15	0.001 (0.002)	0.000 (0.001)	0.53	1.08
**W32**	0.36	0.19	0.12	0.19	0.004	0.001 (0.002)	0.68	1.54
**W33**	1.13	0.44	0.43	0.68	0.011	0.000 (0.001)	2.36	5.06
**W34**	0.38	0.25	0.22	0.31	0.003 (0.004)	0.000 (0.002)	1.15	2.32
**W35**	0.16	0.37	0.23	0.26	0.025	0.002 (0.002)	1.48	2.56
**W36**	0.66	0.84	0.64	0.74	0.005 (0.015)	0.004 (0.010)	3.33	6.23
**W37**	2.17	0.59	0.53	0.76	0.004 (0.005)	0.004	1.64	5.72
**Mean**	0.51	0.46	0.24	0.46	0.02	0.003 (0.002)	1.79	3.48
**SD**	0.51	0.26	0.22	0.36	0.02	0.003 (0.002)	1.11	1.89
**Median**	0.26	0.41	0.18	0.34	0.01	0.000 (0.002)	1.38	2.81
**Min**	0.07	0.14	0.06	0.11	0.002 (0.003)	0.000 (0.002)	0.53	1.08

**Max**	2.17	1.08	1.27	1.58	0.12	0.02	5.62	9.82

Table S 12. Comparison with previous studies for the concentrations of seven PAEs in seawater (ng/L)

Region	DMP	DEP	DiBP	DnBP	BBP	DCHP	DEHP
**Arctic**[11]	n.d.- 0.3	n.d.-0.8	n.d.-0.2	0.008-0.3	n.d.-0.05	-	n.d.-3.3
**North Sea (Germany)**[5]	0.23	1.45	-	1.74	0.07	-	3.8
**North Sea (Belgium)**[12]	-	< 25-753	-	< 5-2645	< 10-343	-	66-766
**East China Sea, China**[13-15]	0.08-830	0.25-2404	5-17256	11-17952	0.09-344	0.05-705	9- 9738
**Bohai and Yellow Seas, China**[16, 17]	n.d.-160	1.18-85.9	89.3-2424	n.d.-2226	n.d.-7.8	1.34-3.12	51.4-3388
**Pearl River Delta, China**[18]	1.7-13.6	＜5.1-34.8	＜30.2-70.3	＜33.2-358	n.d.	n.d.	12.7-1050
**Mediterranean Sea**[19-21]	1.4-140	6.9-870	56.5-383.4	63.4-466	1-100	-	30-5970
**Bay of Biscay, Spain**[22]	7.5±0.4	33±3	-	83±7	8±1	64±4	-
**Barkley Sound, Canada**[23]	-	-	-	18-3000	-	-	10-950
**Puget Sound, USA**[23]	-	-	-	-	-	-	60-640
**Klang River estuary, Australia**[24]	-	-	-	-	-	-	3100-64300
**South Korea**[25]	20-100	20-150	-	40-360	-	-	30-300
**Tropical western Pacific Ocean**[26]	n.d.-7	n.d.-2.1	1.9-14	2.2-13	n.d.-5.5	1.1-7	2.0-9.2
**Caspian Sea, Iran**[27]	490	520	-	-	-	-	-
**Thailand**[28]	-	-	-	230-770	-	-	310-1160
**Tunisia**[29]	-	< 10-17000	< 5-106000	< 29-30500	-	-	< 26-168000
**This work**	0.07-2.17	0.14-1.08	0.06-1.27	0.11-1.58	<n.d.-0.12	<n.d.-0.02	0.53-5.62

Table S 13. Particle-bound fractions (ø) of PAEs in seawater from the South China Sea

Sample	DMP	DEP	DiBP	DnBP	DEHP	PAE
**W01**	0.19	0.20	0.16	0.14	0.51	1.80
**W02**	0.16	0.26	0.24	0.13	0.40	1.90
**W03**	0.06	0.21	0.27	0.13	0.56	2.11
**W04**	0.20	0.35	0.15	0.13	0.71	2.30
**W05**	0.06	0.18	0.23	0.20	0.36	1.71
**W06**	0.14	0.36	0.31	0.24	0.50	2.48
**W07**	0.13	0.19	0.29	0.29	0.33	2.03
**W08**	0.01	0.05	0.07	0.52	0.55	2.14
**W09**	0.06	0.19	0.33	0.30	0.37	1.67
**W10**	0.01	0.07	0.46	0.39	0.16	1.54
**W11**	0.03	0.10	0.54	0.40	0.22	2.71
**W12**	0.02	0.11	0.18	0.15	0.30	2.48
**W13**	0.04	0.09	0.57	0.23	0.17	2.82
**W14**	0.14	0.15	0.21	0.16	0.12	1.26
**W15**	0.05	0.08	0.15	0.08	0.15	0.80
**W16**	0.20	0.22	0.21	0.21	0.21	1.19
**W17**	0.18	0.32	0.15	0.24	0.30	1.62
**W18**	0.15	0.24	0.02	0.16	0.11	0.94
**W19**	0.04	0.02	0.11	0.06	0.16	0.96
**W21**	0.05	0.23	0.47	0.45	0.63	2.65
**W22**	0.06	0.15	0.48	0.36	0.57	2.14
**W23**	0.07	0.21	0.29	0.24	0.47	1.69
**W24**	0.02	0.20	0.22	0.18	0.47	1.72
**W25**	0.01	0.14	0.24	0.19	0.50	1.51
**W26**	0.01	0.12	0.49	0.70	0.45	3.07
**W27**	0.02	0.19	0.26	0.22	0.34	1.50
**W28**	0.06	0.36	0.32	0.38	0.35	2.08
**W29**	0.05	0.33	0.38	0.39	0.29	2.30
**W30**	0.05	0.23	0.28	0.25	0.40	1.74
**W31**	0.04	0.20	0.23	0.18	0.29	1.80
**W32**	0.01	0.20	0.17	0.15	0.28	1.21
**W33**	0.00	0.06	0.04	0.03	0.22	0.56
**W34**	0.04	0.23	0.14	0.14	0.22	1.37
**W35**	0.05	0.15	0.09	0.14	0.14	0.62
**W36**	0.29	0.33	0.23	0.21	0.30	1.74
**W37**	0.02	0.09	0.10	0.06	0.17	1.11
**Mean**	0.08	0.19	0.25	0.23	0.34	1.76
**SD**	0.07	0.09	0.14	0.14	0.16	0.62

Table S 14. Correlation analysis of PAEs in air

	DMP	DEP	DiBP	DnBP	DEHP
**DMP**	1(0.000***)	0.647(0.001***)	0.242(0.279)	0.168(0.455)	0.214(0.339)
**DEP**	0.647(0.001***)	1(0.000***)	0.385(0.077*)	0.398(0.067*)	0.199(0.375)
**DiBP**	0.242(0.279)	0.385(0.077*)	1(0.000***)	0.951(0.000***)	-0.091(0.686)
**DnBP**	0.168(0.455)	0.398(0.067*)	0.951(0.000***)	1(0.000***)	-0.148(0.512)
**DEHP**	0.214(0.339)	0.199(0.375)	-0.091(0.686)	-0.148(0.512)	1(0.000***)

***, **, *means significant 0.01, 0.05, 0.1

Table S 15. Correlation analysis of PAEs in seawater

	DMP	DEP	DiBP	DnBP	DEHP
**DMP**	1(0.000***)	0.659(0.000***)	0.27(0.112)	0.245(0.150)	0.354(0.034**)
**DEP**	0.659(0.000***)	1(0.000***)	0.174(0.311)	0.336(0.045**)	0.55(0.001***)
**DiBP**	0.27(0.112)	0.174(0.311)	1(0.000***)	0.324(0.054*)	0.296(0.080*)
**DnBP**	0.245(0.150)	0.336(0.045**)	0.324(0.054*)	1(0.000***)	0.611(0.000***)
**DEHP**	0.354(0.034**)	0.55(0.001***)	0.296(0.080*)	0.611(0.000***)	1(0.000***)

***, **,*means significant 0.01, 0.05, 0.1

Table S 16. Air-sea exchange fluxes of PAEs (DMP, DEP, DiBP, DnBP and DEHP) in the South China Sea. Positive value indicates water to air volatilization and negative value indicates air to water deposition

F ($ng/m^2/day$)	DMP	DEP	DiBP	DnBP	DEHP
**W01**	-970	-85	-1488	-1015	918
**W02**	-888	-71	-657	-435	57
**W03**	-969	-88	-1502	-1048	419
**W04**	-391	-79	-1278	-899	331
**W05**	-392	-78	-1337	-947	919
**W06**	-243	-147	-671	-489	314
**W07**	-243	-146	-674	-505	528
**W08**	-241	-109	-440	-444	470
**W09**	-245	-110	-434	-335	3538
**W10**	-244	-118	-457	-445	2255
**W11**	-174	-79	-303	-279	1419
**W12**	-176	-86	-313	-297	492
**W13**	-210	-102	-1054	-874	1272
**W14**	-217	-112	-1138	-943	3616
**W15**	-104	-41	-172	-198	733
**W16**	-106	-42	-172	-197	1059
**W17**	-244	-94	-678	-465	444
**W18**	-258	-100	-628	-494	1593
**W19**	-258	-90	-726	-505	601
**W21**	-164	-53	-107	-48	740
**W22**	-181	-161	-959	-634	338
**W23**	-181	-163	-961	-631	600
**W24**	-177	-88	-2080	-1470	310
**W25**	-174	-86	-2042	-1451	214
**W26**	-177	-87	-2178	-1568	233
**W27**	-460	-104	-1349	-1050	435
**W28**	-419	-92	-1120	-927	33
**W29**	-442	-104	-1896	-1579	255
**W30**	-443	-105	-1948	-1622	194
**W31**	-279	-42	-284	-230	159
**W32**	-371	-42	-12	-40	273
**W33**	-1487	-383	-1162	-1500	1308
**W34**	-1497	-391	-1226	-1596	663
**W35**	-44	-21	-328	-229	329
**W36**	-44	-19	-342	-231	1366
**W37**	-190	-77	-406	-326	361
**mean**	-370	-105	-903	-721	800

Table S 17. Particle dry deposition of PAEs in the South China Sea

F_D (ng/m^2/day)	DMP	DEP	DiBP	DnBP	DEHP
**A01**	1.2	3.6	2.2	2.8	94
**A02**	0.9	2.2	16.5	10.9	198
**A03**	15.4	35.7	1396	918	179
**A04**	4.7	12.4	1097	623	85
**A05**	2.8	14.6	877	473	104
**A06**	3.4	6.7	508	590	67
**A07**	1.9	6.5	63	284	68
**A08**	1.8	7.0	165	214	54
**A09**	3.8	12.2	496	454	67
**A10**	2.7	8.0	1378	1135	126
**A11**	2.4	8.3	528	612	92
**A12**	1.4	2.8	53	57	40
**A13**	4.4	5.9	62	83	76
**A14**	3.1	12.5	17	41	86
**A15**	3.5	24.4	553	411	80
**A16**	28.1	24.4	22	29	96
**A17**	2.1	11.0	25	40	103
**A18**	5.0	22.4	53	54	118
**A19**	1.3	3.1	1.6	4.6	26
**A20**	4.7	11.4	634	430	55
**A21**	6.8	34.7	1814	1631	138
**A22**	2.2	5.2	6.4	10	58
**Max**	28	35.7	1814	1631	198
**Min**	0.86	2.21	1.6	2.8	26
**Mean**	4.71	12.5	444	368	91
**SD**	6.04	9.81	548	427	42
**Median**	2.93	9.68	114	249	86

References

1. Bidleman, T. F., Atmospheric processes - wet and dry deposition of organic-compounds are controlled by vapor particle partitioning. *Environmental Science & Technology* **1988,** *22* (4), 361-367.
2. Ligocki, M. P.; Leuenberger, C.; Pankow, J. F., Trace organic compounds in rains-III. Particle scavenging of neutral organic compounds. *Atmospheric Environment* **1967,** *19*, 1619-1626.
3. Bamford, H. A.; Offenberg, J. H.; Larsen, R. K.; Ko, F. C.; Baker, J. E., Diffusive exchange of polycyclic aromatic hydrocarbons across the air-water interface of the Patapsco River, an urbanized subestuary of the Chesapeake Bay. *Environmental Science & Technology* **1999,** *33* (13), 2138-2144.
4. Bamford, H. A.; Ko, F. C.; Baker, J. E., Seasonal and annual air-water exchange of polychlorinated biphenyls across Baltimore Harbor and the northern Chesapeake Bay. *Environmental Science & Technology* **2002,** *36* (20), 4245-4252.
5. Xie, Z. Y.; Ebinghaus, R.; Temme, C.; Caba, A.; Ruck, W., Atmospheric concentrations and air-sea exchanges of phthalates in the North Sea (German Bight). *Atmospheric Environment* **2005,** *39* (18), 3209-3219.
6. Schwarzenbach, R. O.; Gschwend, P. M.; Imboden, D. M., *Environmental Organic Chemistry, 3rd Edition*. 2016.
7. Wanninkhof, R., Relationship between wind-speed and gas-exchange over the ocean. *Journal of Geophysical Research-Oceans* **1992,** *97* (C5), 7373-7382.
8. Gao, Y.; Wang, L. F.; Guo, X. H.; Xu, Y.; Luo, L., Atmospheric wet and dry deposition of dissolved inorganic nitrogen to the South China Sea. *Science China-Earth Sciences* **2020,** *63* (9), 1339-1352.
9. Cousins, I.; Mackay, D., Correlating the physical-chemical properties of phthalate esters using the 'three solubility' approach. *Chemosphere* **2000,** *41* (9), 1389-1399.
10. Perterson, D. R., Staples, C.A., *Degradation of Phthalate Esters in the Environment. The Handbook of Environmental Chemistry*. Springer-Verlag: Berlin/Heidelberg: Berlin, 2003; Vol. 3.
11. Xie, Z. Y.; Ebinghaus, R.; Temme, C.; Lohmann, R.; Caba, A.; Ruck, W., Occurrence and air-sea exchange of phthalates in the arctic. *Environmental Science & Technology* **2007,** *41* (13), 4555-4560.
12. Huysman, S.; Van Meulebroek, L.; Janssens, O.; Vanryckeghem, F.; Van Langenhove, H.; Demeestere, K.; Vanhaecke, L., Targeted quantification and untargeted screening of alkylphenols, bisphenol A and phthalates in aquatic matrices using ultra-high-performance liquid chromatography coupled to hybrid Q-Orbitrap mass spectrometry. *Anal Chim Acta* **2019,** *1049*, 141-151.
13. Zhang, Z. M.; Zhang, J.; Zhang, H. H.; Shi, X. Z.; Zou, Y. W.; Yang, G. P., Pollution characteristics, spatial variation, and potential risks of phthalate esters in the water-sediment system of the Yangtze River estuary and its adjacent East China Sea. *Environ Pollut* **2020,** *265* (Pt A).
14. Wang, L. Y.; Gu, Y. Y.; Zhang, Z. M.; Sun, A. L.; Shi, X. Z.; Chen, J.; Lu, Y., Contaminant occurrence, mobility and ecological risk assessment of phthalate esters in the sediment-water system of the Hangzhou Bay. *Science of the Total Environment* **2021,** *770*, 114913.
15. Zhang, Z. M.; Zhang, H. H.; Zhang, J.; Wang, Q. W.; Yang, G. P., Occurrence, distribution, and ecological risks of phthalate esters in the seawater and sediment of Changjiang River Estuary and its adjacent area. *Sci Total Environ* **2018,** *619*, 93-102.
16. Liu, Y. D.; Li, Z. Z.; Jalon-Rojas, I.; Wang, X. H.; Fredj, E.; Zhang, D. H.; Feng, L. J.; Li, X. G., Assessing the potential risk and relationship between microplastics and phthalates in surface seawater of a heavily human-impacted metropolitan bay in northern China. *Ecotoxicology and Environmental Safety* **2020,** *204*, 111067.
17. Zhang, Z. M.; Zhang, H. H.; Zou, Y. W.; Yang, G. P., Distribution and ecotoxicological state of phthalate esters in the sea-surface microlayer, seawater and sediment of the Bohai Sea and the Yellow Sea. *Environ Pollut* **2018,** *240*, 235-247.

18. Cao, Y. R.; Li, J.; Wu, R. B.; Lin, H. J.; Lao, J. Y.; Ruan, Y. F.; Zhang, K.; Wu, J. X.; Leung, K. M. Y.; Lam, P. K. S., Phthalate esters in seawater and sediment of the northern South China Sea: Occurrence, distribution, and ecological risks. *Science of the Total Environment* **2022,** *811*, 151412.
19. Castro-Jimenez, J.; Ratola, N., An innovative approach for the simultaneous quantitative screening of organic plastic additives in complex matrices in marine coastal areas. *Environ Sci Pollut R* **2020,** *27* (10), 11450-11457.
20. Paluselli, A.; Aminot, Y.; Galgani, F.; Net, S.; Sempere, R., Occurrence of phthalate acid esters (PAEs) in the northwestern Mediterranean Sea and the Rhone River. *Progress in Oceanography* **2018,** *163*, 221-231.
21. Sanchez-Avila, J.; Tauler, R.; Lacorte, S., Organic micropollutants in coastal waters from NW Mediterranean Sea: Sources distribution and potential risk. *Environ Int* **2012,** *46*, 50-62.
22. Prieto, A.; Zuloaga, O.; Usobiaga, A.; Etxebarria, N.; Fernandez, L. A., Development of a stir bar sorptive extraction and thermal desorption-gas chromatography-mass spectrometry method for the simultaneous determination of several persistent organic pollutants in water samples. *J Chromatogr A* **2007,** *1174* (1-2), 40-49.
23. Keil, R.; Salemme, K.; Forrest, B.; Neibauer, J.; Logsdon, M., Differential presence of anthropogenic compounds dissolved in the marine waters of Puget Sound, WA and Barkley Sound, BC. *Mar Pollut Bull* **2011,** *62* (11), 2404-2411.
24. Tan, G. H., Residue Levels of Phthalate-Esters in Water and Sediment Samples from the Klang River Basin. *B Environ Contam Tox* **1995,** *54* (2), 171-176.
25. Heo, H.; Choi, M. J.; Park, J.; Nam, T.; Cho, J., Anthropogenic Occurrence of Phthalate Esters in Beach Seawater in the Southeast Coast Region, South Korea. *Water* **2020,** *12*, 122.
26. Zhang, Q.; Song, J. M.; Li, X. G.; Peng, Q. C.; Yuan, H. M.; Li, N.; Duan, L. Q.; Ma, J., Concentrations and distribution of phthalate esters in the seamount area of the Tropical Western Pacific Ocean. *Marine Pollution Bulletin* **2019,** *140*, 107-115.
27. Hadjmohammadi, M. R.; Fatemi, M. H.; Taneh, T., Coacervative Extraction of Phthalates from Water and Their Determination by High Performance Liquid Chromatography. *J Iran Chem Soc* **2011,** *8* (1), 100-106.
28. Malem, F.; Soonthondecha, P.; Khawmodjod, P.; Chunhakorn, V.; Whitlow, H. J.; Chienthavorn, O., Occurrence of phthalate esters in the eastern coast of Thailand. *Environmental Monitoring and Assessment* **2019,** *191*, 627.
29. Jebara, A.; Albergamo, A.; Rando, R.; Potorti, A. G.; Lo Turco, V.; Ben Mansour, H.; Di Bella, G., Phthalates and non-phthalate plasticizers in Tunisian marine samples: Occurrence, spatial distribution and seasonal variation. *Marine Pollution Bulletin* **2021,** *163*, 111967.
